# Distribution of P1(D1) wart disease resistance in potato germplasm and GWAS identification of haplotype-specific SNP markers

**DOI:** 10.1007/s00122-020-03559-3

**Published:** 2020-02-11

**Authors:** Charlotte Prodhomme, Peter G. Vos, Maria João Paulo, Jasper E. Tammes, Richard G. F. Visser, Jack H. Vossen, Herman J. van Eck

**Affiliations:** 1 Plant Breeding, Wageningen University and Research, Droevendaalsesteeg 1, 6708 PB Wageningen, The Netherlands; 2 Biometris, Wageningen University and Research, Droevendaalsesteeg 1, 6708 PB Wageningen, The Netherlands; 3 Present Address: HZPC Research B.V., Roptawei 4, 9123 JB Metslawier, The Netherlands; 4 Present Address: Averis Seeds B.V., Valtherblokken Z40, 7876 TC Valthermond, The Netherlands

## Abstract

**Key message:**

A Genome-Wide Association Study using 330 commercial potato varieties identified haplotype specific SNP markers associated with pathotype 1(D1) wart disease resistance.

**Abstract:**

*Synchytrium endobioticum* is a soilborne obligate biotrophic fungus responsible for wart disease. Growing resistant varieties is the most effective way to manage the disease. This paper addresses the challenge to apply molecular markers in potato breeding. Although markers linked to *Sen1* were published before, the identification of haplotype-specific single-nucleotide polymorphisms may result in marker assays with high diagnostic value. To identify hs-SNP markers, we performed a genome-wide association study (GWAS) in a panel of 330 potato varieties representative of the commercial potato gene pool. SNP markers significantly associated with pathotype 1 resistance were identified on chromosome *11*, at the position of the previously identified *Sen1* locus. Haplotype specificity of the SNP markers was examined through the analysis of false positives and false negatives and validated in two independent full-sib populations. This paper illustrates why it is not always feasible to design markers without false positives and false negatives for marker-assisted selection. In the case of *Sen1*, founders could not be traced because of a lack of identity by descent and because of the decay of linkage disequilibrium between *Sen1* and flanking SNP markers. *Sen1* appeared to be the main source of pathotype 1 resistance in potato varieties, but it does not explain all the resistance observed. Recombination and introgression breeding may have introduced new, albeit rare haplotypes involved in pathotype 1 resistance. The GWAS approach, in such case, is instrumental to identify SNPs with the best possible diagnostic value for marker-assisted breeding.

**Electronic supplementary material:**

The online version of this article (10.1007/s00122-020-03559-3) contains supplementary material, which is available to authorized users.

## Introduction

Potato wart disease, caused by *Synchytrium endobioticum*, induces the formation of galls on tubers of potato (*Solanum tuberosum*). The pathogen belongs to the phylum of the Chytridiomycota, one of the early diverging fungal lineages. It is an obligate biotrophic soilborne fungus producing winter spores presumably after a sexual phase of its life cycle. These persistent spores can remain viable in the soil for more than 40 years (Przetakiewicz 2015). The yield losses can reach 50 to 100%, and no fungicides are available against this pathogen. Therefore, it has the status of a quarantine disease. When the presence of the fungus is recorded in a field, no potato can be cultivated until soil tests become negative for the presence of sporangia (EPPO 1990). For these reasons, breeding for varieties resistant to the potato wart disease is essential, especially in Europe where the presence of the pathogen has been recorded in many countries (Obidiegwu et al. 2014).

Breeding for potato varieties resistant to *S. endobioticum* is challenging. Before 1941, only pathotype 1 was known and breeding programs produced resistant varieties. After 1941, new pathotypes emerged and most varieties are susceptible to them, because resistance to the available pathotype 1 was evaded (Baayen et al. 2006). The availability of haplotype-specific SNP markers for breeders to screen their clones as well as the potato germplasm to find resistance sources is limited. Consequently, large numbers of laborious disease assays are needed in breeding programmes. Different phenotyping methods exist, consisting either of field trials or laboratory assays using winter (Spieckermann and Kothoff 1924) or summer spores (Glynne 1925; Lemmerzahl 1930) for inoculation. Often, these different methods do not give consistent results, and they are difficult to manage and time-consuming. Thus, molecular markers associated with resistance should be identified for breeding resistant potato varieties.

The dominant monogenic *Sen1* locus confers strong resistance to pathotype P1 (D1), hereafter abbreviated to P1. The *Sen1* locus has been mapped on the north arm of chromosome *11* (Hehl et al. 1999), in a region containing several nucleotide-binding leucine-rich repeat-encoding (NLR) gene clusters. Jupe et al. (2013) described two NLR clusters (C76 and C77) at this position of chromosome *11*. Both clusters consisted of Toll/interleukin-1 receptor nucleotide-binding leucine-rich repeat (TNL) encoding genes. Another dominant monogenic locus conferring P1 resistance, *Sen1*-*4*, has been mapped to the south arm of chromosome *4* (Brugmans et al. 2006). Additional genetic studies have been performed using diploid and tetraploid populations to identify wart disease resistance loci. *Sen2* is a dominant monogenic locus, bringing strong resistance to a wide range of pathotypes, located on chromosome *11* approximately 32 Mb south from *Sen1* (Plich et al. 2018). *Sen3*, another dominant monogenic locus involved in strong resistance to pathotypes 2, 6 and 18, was fine-mapped to the same region as *Sen1* (Bartkiewicz et al. 2018; Prodhomme et al. 2019). Multiple QTLs involved in resistance to several pathotypes or in pathotype-specific resistance were also identified across the potato genome in different genetic backgrounds (Ballvora et al. 2011; Groth et al. 2013; Obidiegwu et al. 2015). Nevertheless, few of the markers linked to *Sen1* or these other monogenic loci and QTLs have been validated in a wider panel of distantly related varieties, representative for the entire gene pool of commercial potato.

Such a validation experiment has been performed for markers linked to P1 resistance by Obidiegwu et al. (2015), but the markers they tested were, inconveniently, linked in repulsion phase with the resistance locus. The Nl25 marker, used for the first mapping of *Sen1* (Gebhardt et al. 2006; Hehl et al. 1999), was recently validated in a limited set of twelve resistant and susceptible varieties by Przetakiewicz and Plich (2017) and in a panel composed only of Russian varieties (Antonova et al. 2017). Nl25, GP125 and Stl046, all found to be linked to *Sen1* in mapping populations, were screened in a panel of 89 Polish and German varieties in the CORNET project SynTest, but the results were not elaborately reported (Bartkiewicz et al. 2018). The two DArTseq markers flanking *Sen1* designed by Plich et al. (2018) in a diploid segregating population were not validated in tetraploid varieties.

Compared to traditional linkage mapping approaches, genome-wide association studies (GWAS) allow to include the genetic diversity of a wider panel of varieties and to reach a superior mapping resolution thanks to a higher number of recombination events. Aside from studies in genetic model organisms, GWAS has already been successfully used in several crops such as soybean (Bandillo et al. 2015), rice (Zhao et al. 2011), wheat (Sukumaran et al. 2015) as well as potato (D’hoop et al. 2014; Uitdewilligen et al. 2013). GWAS has never been applied to identify loci involved in *S. endobioticum* resistance, although resistance values are well documented for varieties on National Lists motivated by the Value for Cultivation and Use (VCU) requirements.

In this study, we performed a GWAS in a panel of 330 tetraploid potato varieties representative of the gene pool of commercial potato breeding. We identified a region with SNPs strongly associated with pathotype 1 resistance on the north arm of chromosome *11*. Four significantly associated SNP markers were selected and validated in two different biparental populations segregating for P1 resistance, to demonstrate their linkage in coupling phase with the *Sen1* locus. The markers do not explain all the P1 resistance present in the GWAS panel. This suggests a more complex architecture of pathotype 1 resistance involving one predominant gene with a major effect along with other less frequent haploblockswith *Sen1* or *Sen1*-like alleles, or loci beyond chromosome *11*.

## Materials and method

### GWAS panel

A subset of the panel of 569 potato genotypes described in Vos et al. (2015) was used, because phenotypic data for wart disease were available for 330 individuals. These individuals are representative for the gene pool of commercial potato, because it includes old and recent varieties, developed for different markets, widely used progenitors, as well as Dutch advanced breeding lines (Supplementary File 1). Because this panel is composed of 304 commercial varieties and only 26 progenitors and breeding clones, hereafter we use the term of varieties for this GWAS panel.

### GWAS panel genotyping data

The genotyping data used for GWAS were obtained by Vos et al. (2015) with a 20K Infinium SNP array (hereafter mentioned as the SolSTW array) comprising 4463 attempted SNPs from the potato SolCAP array (Felcher et al. 2012; Hamilton et al. 2011) and 16.5K attempted PotVar SNPs from a targeted enrichment study (Uitdewilligen et al. 2013). For 14,530 SNPs in the panel, the assay quality allowed determination of the tetraploid scores of the markers, with a score of 0 for a nulliplex genotype call, 1 for simplex, 2 for duplex, 3 for triplex and 4 for quadruplex. Markers were removed when missing values exceeded 20%, when the minor allele frequency (hereafter MAF) was below 1.25%, and when the markers were present in less than 6 varieties, resulting in a subset of 10,968 SNPs used for the GWAS (Supplementary File 1). The MAF threshold of 1.25% implies for a tetraploid species that ~ 5% of the varieties have this SNP allele in simplex condition, which is equivalent to the commonly used threshold of 5% in diploids.

### Population structure

The population structure of the variety panel was studied using a kinship matrix, calculated using VanRaden (2007), with a random subset of 1000 markers (Supplementary File 1) to perform a principal coordinate analysis (PCoA), which suggested four structure groups. STRUCTURE (Pritchard et al. 2000) was used to assign each variety to one of the four groups. We used the settings advised by Evanno et al. 2005: length of burn-in period 10,000, number of MCMC reps after burn-in 10,000, admixture model, allele frequencies correlated among the populations. We made 10 repetitions for *K* = 4 (Supplementary File 2).

### GWAS panel phenotypic data

Phenotypic data for P1 resistance were collected from various sources, such as National Lists (Value for Cultivation and Use (VCU data)), various websites and brochures from commercial breeders (Supplementary File 3). As the scales used by the different sources varied, we converted the scales into resistant scores ranging from 1 (highly susceptible) to 10 (highly resistant), as described in Supplementary File 3. During a preliminary GWAS, false-positive varieties were identified (susceptible varieties holding P1-associated markers). For some of these varieties, the phenotypic data we gathered were scarce or inconsistent. Therefore, for 12 varieties, new disease assays were performed to assess these inconsistencies. The reassessment was performed during spring 2016 by HLB (Wijster, the Netherlands) using both the Spieckermann (Spieckermann and Kothoff 1924) and Glynne–Lemmerzahl (Glynne 1925; Lemmerzahl 1930) methods with 9 and 5 tuber eye pieces, respectively. Isolate MB42 P1(D1) was used for both tests (van de Vossenberg et al. 2018). The same quantitative scoring scale was used for both experiments, ranging from 10 (highly resistant, corresponding to type 1 in Germany and type—in the Netherlands) to 1 (highly susceptible, corresponding to type 5 in Germany and type X in the Netherlands). Varieties rated in a category lower than 8 are considered as susceptible as it corresponds to very late defence necrosis, which can be slower than the pathogen maturation (EPPO 2004). For each variety assessed, a mean score was calculated (Supplementary Table 1). Hence, the final phenotypic data set used in the rest of the analysis included corrected phenotypes for these 12 varieties.

### Genome-wide association study

The genotypic data ranged from 0 to 4 reflecting the dosage of the SNPs. For each variety in the panel, we calculated a final resistance score corrected for the source effect using restricted maximum likelihood (REML). After correction for data source, phenotypes ranged from 0.85 to 10.72. The model included the source as a random effect and the variety as a fixed effect as follows: ${\text{Pathotype}}1\;{\text{resistance}} = {\text{source}} + {\text{variety}}$. We carried out the association analysis using three different association models. Firstly, we use a simple linear regression model without any structure correction (naive model). Secondly, we use the 30 first principal coordinates of the PCoA performed on the VanRaden kinship as a fixed effect in a mixed linear regression model to correct for the population structure. In the third model, we included five cofactors (most significant markers from the PCoA corrected model) as fixed effects in the PCoA corrected model. Quantile–quantile (qq) plots were produced to compare the models. The GWAS models were fitted in GenStat version 18 (VSN International 2015). The genome-wide significance threshold – log_10_(*p*) = 4.7 was calculated by procedure QTHRESHOLD, using the method developed by Li and Ji (2005). GWAS results were visualised on R version 3.2.2 using the EasyStrata package (Winkler et al. 2015).

### Validation populations

Populations segregating for P1 resistance were used to validate markers identified by the GWAS. The P1-resistant varieties Desiree and Kuras were crossed with Aventra (susceptible to P1) giving 42 (A × D population) and 35 progeny (K × A population), respectively. The descendants were phenotyped for their resistance to P1 during spring 2016 with the Spieckermann (Spieckermann and Kothoff 1924) and the Glynne–Lemmerzahl (Glynne 1925; Lemmerzahl 1930) methods with 8 and 6 eye pieces, respectively. Two subsets of 9 and 17 descendants from the A × D and K × A populations were re-phenotyped in spring 2017 with 12 and 6 eye pieces for the Spieckermann and the Glynne–Lemmerzahl methods, respectively. The Spieckermann assays were performed by HLB using isolate MB42. The Glynne–Lemmerzahl assays were performed by the Laboratory of Quarantine Organisms, Department of Plant Pathology, IHAR-PIB, Poland, with the isolate JKI P1(D1)-2009. Disease symptoms were rated from 1 (highly resistant, early defence necrosis) to 5 (highly susceptible). Mean scores were calculated between replicates (6–8 eye pieces, or 6–12 tubers) within years and between the two assessment years.

### DNA extraction

Total genomic DNA was isolated from freshly harvested freeze-dried tubers or fresh leaves using a modified CTAB method (Fulton et al. 1995). The DNA concentration was estimated using a NanoDrop ND-1000 spectrophotometer (Thermo Scientific) and adjusted to a concentration of 5–50 ng/μl. The DNA quality was assessed on ethidium bromide containing agarose gels.

### SNP validation

To validate linkage in coupling phase of significantly associated SNPs, Kompetitive Allele Specific Polymorphisms (KASP) markers were designed. KASP primers design was done by LGC Genomics (LGC, Hoddeston, UK) or in-house, using Primer3 (v3.0.0) (Rozen and Skaletsky 2000). All KASP primers are given in Supplementary Table 2. Segregations patterns and associations between markers and P1 resistance were tested using Chi-square tests and Kruskal–Wallis tests, respectively. All statistical analyses were performed with R version 3.2.2.

## Results

### GWAS panel structure and P1 resistance data

**Fig. 1:**
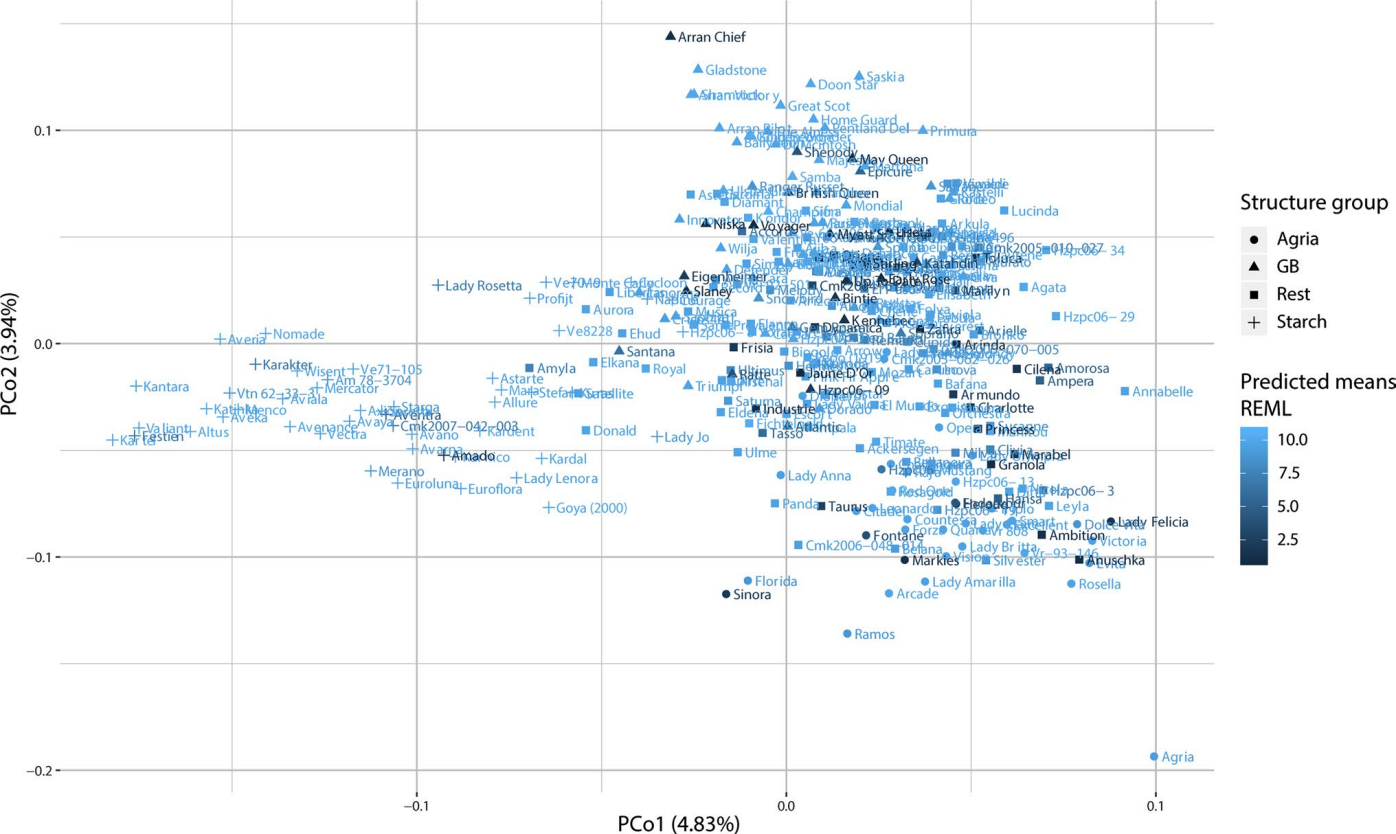
Structure of the panel of 330 potato varieties was analysed using the first two principal coordinates of the PCoA performed on a random subset of 1000 SNPs on the GWAS panel. Each variety is represented by a pseudo-colour that scales with the level of resistance to P1 (dark colour (P1 predicted mean < 6) indicates highly susceptible varieties). The shapes of the dots indicate the STRUCTURE group affiliation of each variety according to Supplementary File 2

A panel of 330 tetraploid potato varieties, composed of old widely used clones as well as recent varieties from 14 different countries, was used for GWAS (Vos et al. 2015; Supplementary File 1; Supplementary Fig. 1). A principal coordinate analysis and a STRUCTURE analysis were performed on a random subset of 1000 SNPs (Fig. 1). The first two principal coordinates (PCo) explained 8.8% of the variance and showed four groups in the GWAS panel. The first group (‘starch’) was composed of varieties bred for starch industry. The second group (‘GB origins’) was composed of varieties with British origins. The third group (‘Agria’) has varieties descending from Agria. Agria, at the edge of this cluster, was frequently used in the 1990s to breed for varieties suitable for French fries. The last group (‘rest’) was composed of varieties with diverse origins.

The phenotypic values for P1 resistance in the GWAS panel showed a bimodal distribution, skewed towards resistance (Supplementary Fig. 1). The skewed distribution reflects the historical progress in breeding wart-resistant varieties. Before the 1920s, 50% of the varieties were resistant to P1, whereas since the 1980s more than 77% of the varieties are resistant. In the ‘starch’ structure group, most varieties were resistant (Supplementary Fig. 2). Only Amado, Aventra, CMK2007-042-003, Festien, Karakter and Lady Rosetta were susceptible (scores below 8). Resistant varieties were also predominant in the ‘Agria’ structure group, which contained only seven susceptible varieties (Endeavour, Fontane, Heroud, Hzpc06-11, Lady Felicia, Markies and Sinora). The group containing varieties with British origin showed most variation for resistance: 31% of the varieties from this group were susceptible. These observations suggested a correlation between the panel structure and potato wart resistance. Indeed, the 30 first principal coordinates of the PCoA were 51% correlated with P1 resistance and were consequently included in the GWAS model to correct for the structure confounding effect.

In view of the skewed distribution of phenotypic values, the distribution of the residuals of the PCoA corrected GWAS including the five most significant but uncorrelated markers as cofactors was examined. The close to normal distribution of residuals confirmed the legitimacy of using a linear regression model for GWAS (Supplementary Fig. 3).

### Genome-wide association study of P1 resistance

**Fig. 2:**
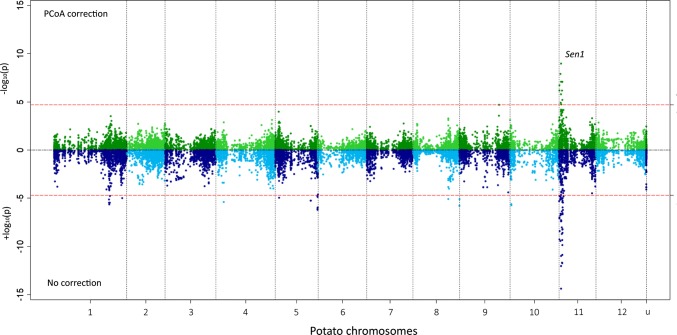
Miami plot of the GWAS of pathotype 1 resistance without the PCoA correction (in blue, lower part of the plot) and with the PCoA correction (in green, upper part of the plot) performed on 330 potato varieties. The association (± log_10_(*p*)) of each SNP with pathotype 1 resistance is represented. The x axis represents the physical position of each SNP on the 12 potato chromosomes (u corresponds to markers of unanchored regions and to chloroplast markers). The red horizontal lines correspond to the significance threshold calculated with the Li and Ji method (− log_10_(*p*) = 4.7). The strongly associated region at the beginning of chromosome *11* corresponds to the *Sen1* locus previously identified by Hehl et al. (1999)

Association between phenotypic values for P1 resistance of 330 tetraploid varieties and 10,968 SNP markers with the naive model identified a clear pileup of 47 SNPs on chromosome *11*. In addition, 23 SNPs from several other genomic regions showed significant association with wart resistance. However, when using the PCoA corrected model, only 12 markers remained associated with P1 resistance, all located on the north arm of chromosome *11* between 0.78 Mb and 4.35 Mb (Fig. 2, Table 1). This region is known to harbour the *Sen1* locus associated with P1 resistance (Hehl et al. 1999).

**Table 1:** Significantly associated markers in the PCoA corrected GWAS model

SNP	Chromosome	Position (bp)	*P* value	Effect	MAF	FPR	FNR
PotVar0064472^a^	11	787,356	6.66E−07	1.04	0.41	0.18	0.06
solcap_snp_c2_13431^a^	11	788,222	1.92E−07	1.08	0.41	0.18	0.06
PotVar0066243^a^	11	2,060,973	1.28E−08	1.04	0.48	0.19	0.01
PotVar0066295^a^	11	2,261,174	1.23E−05	0.78	0.49	0.20	0.02
PotVar0066434^b^	11	2,704,904	1.63E−06	− 0.84	0.39	0.22	0.12
PotVar0067008^c^	11	2,773,680	1.07E−09	1.47	0.17	0.04	0.24
PotVar0067303^a^	11	2,780,260	8.20E−08	0.97	0.36	0.15	0.06
PotVar0067483^a^	11	3,074,626	1.84E−05	0.86	0.26	0.11	0.15
solcap_snp_c1_2314^c^	11	3,928,601	6.78E−07	1.23	0.18	0.06	0.23
PotVar0106272^c^	11	4,224,342	8.42E−08	1.32	0.18	0.06	0.23
PotVar0106247^c^	11	4,227,848	8.52E−08	1.26	0.19	0.08	0.23
PotVar0105904^c^	11	4,348,897	6.33E−06	1.12	0.18	0.06	0.25

Chromosome number, PGSC v4.03 coordinate (PGSC 2011), significance, the effect of a minor allele substitution in the GWAS panel and its allele frequency (MAF). For each SNP, the false-positive rate (FPR) and the false-negative rate (FNR) are given

^a^SNPs with a high FPR and a high MAF: not haplotype specific

^b^SNP with a negative effect

^c^SNPs with a lower FPR and a lower MAF: higher haplotype specificity

With a QQ plot, the corrected and uncorrected (naive) models were compared to study *p* value inflation (Supplementary Fig. 4). Without PCoA correction, the distribution of the observed –log_10_(*p*) values deviated from the diagonal line represents the expected values under a model of no association. The model including the 30 first PCo effectively decreased this deviation. This justifies the conclusion that other regions identified with the naive model are false positives.

### Analysis of predictions of SNPs from chromosome *11*

For each of the 12 associated SNPs from chromosome *11* in the PCoA corrected results, we calculated the false-positive rate (FPR) and the false-negative rate (FNR). The FPR is the percentage of varieties which hold the marker allele but are susceptible, whereas the FNR is the percentage of resistant varieties that lack the marker allele. According to the MAF, the FPR and the FNR, we divided the associated SNPs into three groups. The first group, located between 0.78 and 3.07 Mb, contained six SNPs (PotVar0064472, solcap_snp_c2_13431, PotVar0066243, PotVar0066295, PotVar0067303 and PotVar0067483; Supplementary File 1, Table 1) with a high frequency in the GWAS panel (0.26 ≤ MAF ≤ 0.49), a high FPR between 11 and 20% and a low FNR between 1 and 15%. These SNPs descended from heirloom varieties such as Yam (< 1787), Myatt’s Ashleaf (1804), Jaune d’Or (< 1850) and Pink Fir Apple (1850), of which Myatt’s Ashleaf and Pink Fir Apple are known to be resistant. The raw data (Supplementary File 1) do not suggest that the markers from group 1 are indicative for a specific haplotype conferring resistance in spite of their significant association with wart disease resistance and cannot be recommended for marker-assisted selection (MAS). The second group contained one marker (PotVar0066434) with a negative effect on resistance. This marker, presumably linked in repulsion phase to P1 resistance, is not useful for MAS either. The third group had five markers (PotVar0067008, solcap_snp_c1_2314, PotVar0106272, PotVar0106247 and PotVar0105904), located more south on chromosome *11* (2.77–4.35 Mb), with an MAF of ~ 18%. These SNPs had a low FPR (0.04 ≤ FPR ≤ 0.08) but a higher FNR (0.23 ≤ FNR ≤ 0.25). Based on the year of market introduction of the variety, all group 3 markers are historically first observed in Pink Fir Apple and absent in older varieties. The pattern of marker raw data suggests that these markers are in strong LD and belong to a haplotype that includes resistance to P1, albeit historical recombination events led to decay of LD, proportional to the distance of the markers from the resistance gene (Vos et al. 2017).

### Phenotypic analysis of resistance in two validation populations

To further validate the significantly associated SNPs, we tested two tetraploid full-sib populations Aventra (S) × Desiree (R) and Kuras (R) × Aventra (S) with the Spieckermann and the Glynne–Lemmerzahl method (Supplementary Fig. 5, Supplementary File 4). Resistance scores obtained with the two phenotyping methods were significantly correlated (*R*^2^ = 0.5, *P* value < 0.0001). With the Spieckermann method, the offspring of Desiree and Kuras were comparable in their transmission and level of resistance to P1 (Kruskal–Wallis test, *P* value = 0.16). With the Glynne–Lemmerzahl method, the Aventra × Desiree population showed a higher level of resistance than the Kuras × Aventra population (Kruskal–Wallis test, *P* value = 0.023). The Spieckermann method showed transmission of P1 resistance skewed towards resistance: 81% and 68.6% of the Aventra × Desiree and Kuras × Aventra populations were resistant, respectively. The Glynne–Lemmerzahl method, however, showed a 1:1 segregation ratio (Chi-square test *P* value = 0.3) suggesting a monogenic major effect locus. The P1 segregation analysis with Spieckermann data was flawed due to escapes and does not warrant to postulate a different genetic model which accommodates for differential recognition of factors from either summer or winter spores.

### Genotypic analysis of two validation populations with SNPs markers

**Fig. 3:**
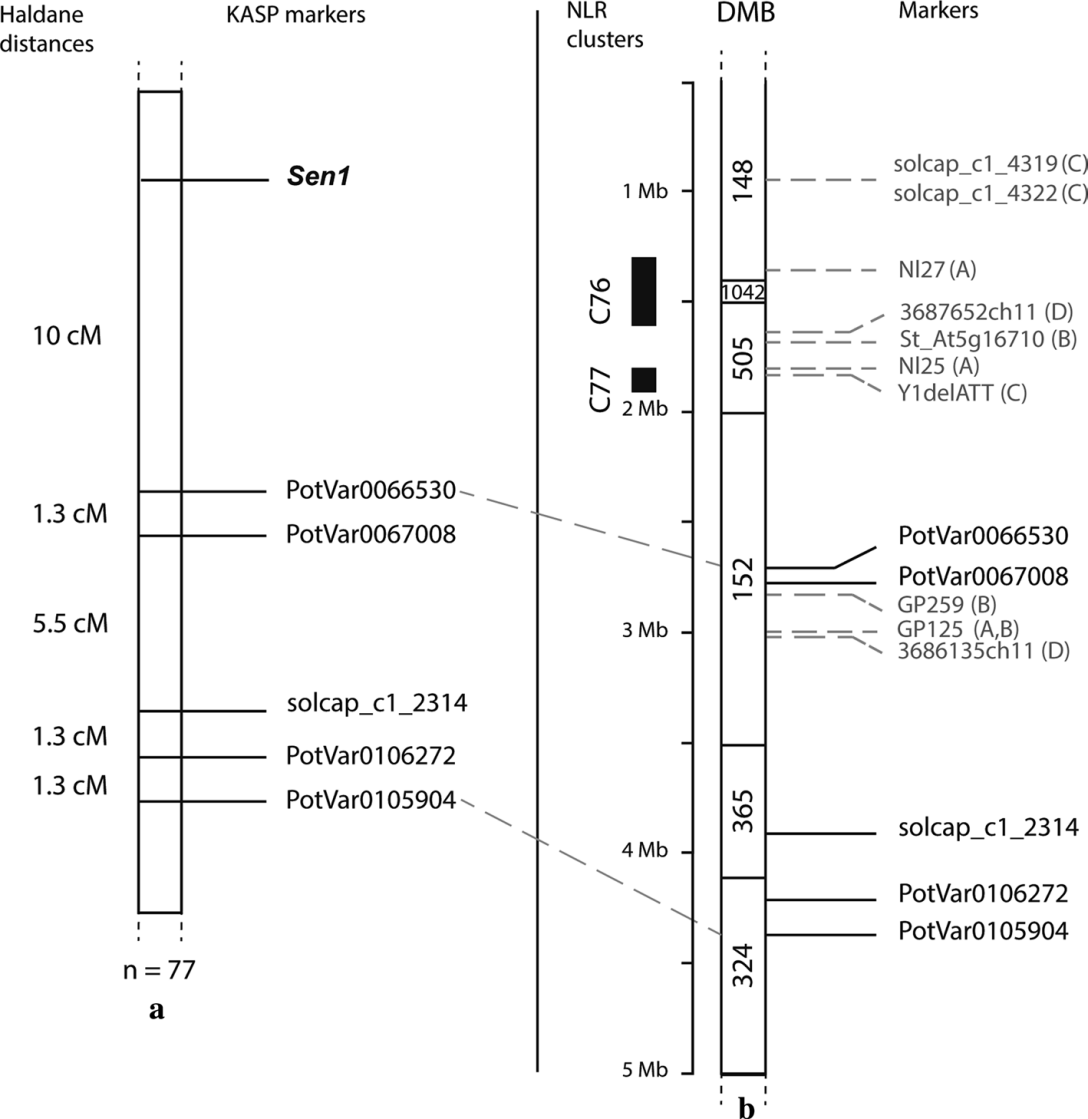
Genetic and physical maps comprising the *Sen1* locus. **a** Consensus genetic map of the *Sen1* haplotype in the combined AD and K × A validation populations (*n* = 77). The Glynne–Lemmerzahl phenotypic data were used to build the map. **b** Physical map of the chromosome *11* north arm adapted from Prodhomme et al. (2019). The KASP markers tested in this study are anchored on the right according to their physical position on DM v4.03 (Potato Genome Sequencing Consortium et al. 2011) and on the left are the NLR clusters according to Jupe et al. (2013). In grey are the markers designed in previous mapping studies: **A** Hehl et al. (1999), **B** Ballvora et al. (2011), **C** Obidiegwu et al. (2015), **D** Plich et al. (2018)

KASP markers were designed for SNPs that were significant in the PCoA corrected GWAS model. We chose four of the five SNPs from group 3 that showed a lower MAF, a lower FPR and a higher FNR. The minor allele of these SNPs was present in simplex in Desiree and Kuras and absent from Aventra according to the SolSTW array data (Supplementary File 1). The markers were screened in the two populations, and their segregation fitted the expected 1:1 segregation ratio (Chi-square tests, *P* values > 0.05) (Supplementary File 4). In the A × D population, none of the markers was associated with the phenotypic data collected with the Spieckermann assays (Table 2). However, all the markers were strongly associated with P1 resistance using the Glynne–Lemmerzahl assays. In the K × A population, the four markers cosegregated with P1 resistance collected with both assays (Fig. 3).

**Table 2:** Marker validation in Aventra × Desiree and Kuras × Aventra offspring. Kruskal–Wallis tests indicate the association of the markers with pathotype 1 resistance in each population and for the Spieckermann and the Glynne–Lemmerzahl phenotyping method

Marker	PGSC^a^ chr11	Aventra × Desiree		Kuras × Aventra	
		Spieckermann	Glynne–Lemmerzahl	Spieckermann	Glynne–Lemmerzahl
PotVar0067008	2,773,680	0.06	1.14E−04***	1.67E−06***	3.93E−07***
solcap_snp_c1_2314	3,928,601	0.20	1.87E−03**	2.70E−05***	1.28E−05***
PotVar0106272	4,224,342	0.17	2.12E−03**	1.67E−06***	7.06E−06***
PotVar0105904	4,348,897	0.12	4.53E−03**	2.50E−06***	1.16E−05***

For each method, the resistance scores across 2016 and 2017 were averaged

**P value < 0.01

***P value < 0.001

^a^Physical coordinates (in bp) of the markers on the potato reference genome (v.4.03)

### Graphical genotyping to identify additional SNPs from the *Sen1* haplotype

PotVar0067008 was fully cosegregating with *Sen1* in the K × A population, but there were eight recombinant events between the marker and *Sen1* in the A × D population (Fig. 3, Supplementary File 4). To develop KASP markers closer to the NLR clusters C76 and C77 (Jupe et al. 2013), we used the 135 K PotVar SNP data set developed by Uitdewilligen et al. (2013) from which markers on the SolSTW SNP array were selected. By graphical genotyping (Van Eck et al. 2017; operators used: allele frequency between 12 and 24%, absent in Black 1256, Ackersegen, Y 66-13-636 and Yam, but present in Arran Pilot), we could identify a haploblock comprising 54 SNPs including PotVar0067008 (Supplementary Fig. 6). As this haploblock ranged from 2.70 to 2.78 Mb, we decided to design a KASP assay for a most distal SNP, PotVar0066530 located at 2,706,497 Mb. The marker was indeed cosegregating with resistance, but there were still seven recombinants (Fig. 3; Supplementary File 4). With operators: MAF > 30%, absent in susceptible varieties Bintje, Industrie and Arran Chief, but present in resistant Arran Pilot, 31 SNPs were identified in high LD with PotVar0066243 from coordinates ranging from 2.03 to 2.26 Mb, but no haplotype structure was observed that would allow to develop additional KASP markers to reduce the amount of false positives or false negatives (Supplementary Fig. 7).

### *Sen1* distribution and contribution to P1 resistance

In the full-sib populations, the presence of the *Sen1* markers significantly increased the resistance to P1. With the Spieckermann phenotyping method, the P1 resistance scores increased from an average of 7.25 to 9.93 in the combined populations when the minor allele of PotVar0066530 was present (Supplementary Fig. 8). With the Glynne–Lemmerzahl method, the average score decreased from 3.6 to 1.4. In the A × D offspring, 58.8% and 79.2% of the resistant descendants with the Spieckermann and the Glynne–Lemmerzahl methods, respectively, held the minor allele of PotVar0066530 (Supplementary Fig. 5). In the K × A population, 78.3% and 100% of the resistant varieties with the Spieckermann and Glynne–Lemmerzahl assays, respectively, held the minor allele of the marker. The difference between the Spieckermann and the Glynne–Lemmerzahl results observed in both populations might be explained by escapes in the Spieckermann assays. The A × D offspring showed a higher proportion of resistant progeny which did not hold the minor allele compared to the K × A population. This could be explained by a higher number of recombinants between PotVar00066530 and *Sen1* or by the fact that another allele involved in P1 resistance segregated in this population.

In the GWAS panel, the average P1 resistance score was 7 when the minor allele of PotVar0067008 was absent, while the scores were 9.5, 9.9 and 9.5 when the minor allele was present in simplex, duplex and triplex, respectively (Supplementary Fig. 8), which is in agreement with a dominant effect without additivity. The number of susceptible varieties and the variance of the P1 resistance scores decreased with the higher dosages, which can be explained by the fact that a variety is less likely to inherit several recombined haplotypes. Overall, the PotVar0067008 minor allele was present in 68% and 16% of the resistant and susceptible varieties of the GWAS panel, respectively. The false negatives were probably not only due to recombination events between *Sen1* and PotVar0067008 but also to other alleles and/or other loci involved in P1 resistance present in the GWAS panel.

The minor allele of PotVar0067008 could be observed in every structure group of the GWAS panel (Supplementary Fig. 9). It was highly frequent in the ‘Agria’ and in the ‘starch’ structure groups: 74.4% and 74.5% of the two groups, respectively, hold the minor allele. In contrast, only 56.4% and 35.1% of the ‘Rest’ and the ‘GB’ groups, respectively, hold the minor allele of PotVar0067008. Selection for *Sen1* containing varieties was very successful in the starch structure group as almost 20% of the varieties hold the minor allele in duplex.

### Other P1 resistance sources are available in breeding germplasm

To identify putative other P1 resistance loci present in the gene pool, we added five mutually uncorrelated, but significantly associated markers from the chromosome *11* peak as a cofactor with fixed effect in the PCoA corrected model (PotVar0067008, PotVar0067303, PotVar0066243, SolCAP_snp_c2_13431 and Potvar0066434). The signal of the chromosome *11* peak was reduced due to the inclusion of these cofactors (Supplementary Fig. 10). Only one marker, PotVar0067876, remained significant considering the Li and Ji association threshold (Supplementary Table 3). This marker was not significant in the two previously applied models (-log_10_(*p*) = 0.61 in the naive model, -log_10_(*p*) = 0.009 in the PCoA corrected model) and had a relatively high FPR and FNR of 16.36% and 20.3%, respectively. This marker might reveal the existence of another haplotype on chromosome *11* involved in quantitative P1 resistance. This hypothesis would require validation.

We performed a new PCoA on the 144 varieties from the GWAS panel which do not contain the minor allele of PotVar0067008 (Supplementary Fig. 11). The ‘Agria’ structure group observed in the entire panel is underrepresented in this PCoA as most (74.4%) of the ‘Agria’ varieties hold the minor allele of PotVar0067008 (Supplementary Fig. 7). A separate cluster of ‘starch’ varieties is observed without PotVar0067008 but resistant to P1 (except for Aventra). This could imply the presence of other wart resistance sources than *Sen1* in the ‘starch’ group. Likewise, the ‘GB’ varieties are a clear group, composed of 27 resistant and 23 susceptible varieties. Again, this could imply the presence of other wart resistance sources in this structure group. In the 71 varieties from the ‘rest’ group, 39 showed resistance to P1. For this group, it is less obvious to make assumptions about other wart resistance sources.

## Discussion

### Successful identification of *Sen1* using GWAS

Pre-existing phenotypic data on resistance to potato wart disease P1 have been collected from various public and private sources for 330 potato varieties. The phenotypes were associated with previously generated SNP data (Vos et al. 2015). Analysis of population showed four groups: starch varieties, processing varieties descending from Agria, varieties of British origin and a miscellaneous group. Because the population structure correlated with P1 resistance, we applied a PCoA correction in our GWAS model to correct for the structure confounding effect. The GWAS resulted in one peak of 12 strongly associated SNPs on the north arm of chromosome *11* where the *Sen1* locus was mapped previously. We could divide these associated SNPs into three groups: (1) common SNPs with a high FPR lacking haplotype specificity, (2) one marker with a negative effect on resistance, probably linked in repulsion phase with *Sen1,* and (3) markers showing a low FPR and lower MAF in the panel. The markers from the last group belonged to a resistant haplotype first observed in Pink Fir Apple. We designed KASP markers for four SNPs from the last group and screened them in two independent biparental populations. In the Aventra × Desiree population, a 10 cM distance was found between *Sen1* and PotVar0066530 (at PGSC coordinate Chr11: 2.706.497), but no recombinant was found in the Kuras × Aventra population. As all recombinants were found among resistant offspring, escapes from the disease assays are likely erroneously regarded as recombination events.

### The Glynne–Lemmerzahl method is less ambiguous than the Spieckermann method

Our mapping populations were phenotyped with the two commonly used methods recommended by the EPPO (EPPO 2004). Their main difference is the material used as inoculum: compost with winter sporangia (Spieckermann and Kothoff 1924) versus fresh warts containing both winter and summer sporangia (Glynne 1925; Lemmerzahl 1930). Although both methods correlate well, the phenotypic distribution obtained with the Spieckermann method was skewed towards resistance, suggesting 15 escapes among 77 descendants. Few studies have compared both methods. Przetakiewicz and Kopera (2007) reported that the Spieckermann method is cheaper and less laborious than the Glynne–Lemmerzahl method, but the lower infection pressure and can lead to escapes. Escapes can be avoided in the Spieckermann method when more tubers are tested.

### *Sen1* probably resides in the C76 or C77 TNL gene cluster

Our results showed that *Sen1* is located north to PotVar0066530 (2.7 Mb), where the C77 and C76 TNL clusters are located (Jupe et al. 2013). Here, the *Sen3* gene has been mapped as well (Bartkiewicz et al. 2018; Prodhomme et al. 2019). However, speculations about the physical position of *Sen1* relative to the DM reference genome should be taken carefully. The reference genome has two gaps, one of which should contain the unanchored scaffold DMB734 (Prodhomme et al. 2019). In a first report, Hehl et al. (1999) mapped *Sen1* distal to Nl25 and Nl27 which is in agreement with our results (Fig. 3). In subsequent reports (Ballvora et al. 2011; Groth et al. 2013; Obidiegwu et al. 2015), no markers flanking *Sen1* were designed, but markers associated with P1 resistance were identified. When the physical position of these markers on the potato reference genome was extrapolated, they pointed to the same region as identified in the current study (Fig. 3). In the diploid population in which the gene *Sen2* was identified, *Sen1* was mapped between 1.64 Mb and 3.08 Mb which means *Sen1* would more likely belong to the C77 TNL cluster than to the C76 TNL cluster (Jupe et al. 2013). However, the mapping results of this paper are different from the mapping results of Hehl et al. (1999) as they mapped *Sen1* proximal to Nl25 and Nl27. According to Plich et al. (2018), this different position could be explained by the different genetic background or by the type of markers which were used in the two different studies.

### A historical interpretation of *Sen1* distribution in potato germplasm

The oldest variety from the panel in which PotVar0067008 was observed was Pink Fir Apple (released in 1850, pedigree unknown), but this variety has hardly contributed to the contemporary gene pool. Pink Fir Apple is duplex for PotVar0067008, but this does not imply that this variety is duplex for two identical haplotypes, or two copies of *Sen1*. This study largely illustrates that such historical reconstructions of the breeding history of haploblocks with the *Sen1* resistance locus are complicated. We assume that related varieties or unnamed landraces with resistance should have existed, as PotVar0067008 is also present in the resistant varieties Champion (1876) and Belle De Fontenay (1885), both unrelated to Pink Fir Apple. These findings suggest that *Sen1* was already present in the gene pool before *S. endobioticum* was first described in Europe in the 1880s (Gough 1919; Hampson 1993). These observations would argue for at least one minor allele being present in early material grown in Europe. However, breeders may have introgressed additional *Sen1* allele(s) or paralogs of *Sen1* along with other *R* genes on the north arm of chromosome *11* such as *Solanum andigena* (*Ry*_*adg*_) (Hämäläinen et al. 1997) and *Solanum stoloniferum* CPC 2093 (*Ny(o,n)*_*sto*_) (van Eck et al. 2017). Unfortunately, pedigree analysis did not allow to identify a common ancestral donor for the *Sen1* locus. This is in contrast to the more recently introgressed *Sen3* (Prodhomme et al. 2019) where all cases of resistance are identical by descent.

About 37% of the varieties from the GWAS panel, registered before the 1960s, hold *Sen1*, against 60% of the varieties registered after 1960. These numbers illustrate well how successful the breeders were in selecting for P1 resistance in their breeding programs. Indeed, varieties such as Abundance, Flourball, Irish Cobbler, Jubel and Majestic were used in many crosses as P1-resistant parent (Black 1935; Lunden and Jørstad 1934; Weiss 1925), albeit Irish Cobbler does not contain PotVar0067008. In the GWAS panel, we observed that *Sen1* was especially selected in the starch and processing potatoes programs, where ~ 75% of the varieties from the ‘starch’ structure group have *Sen1,* including 20% in two copies. These observations make sense, as starch potatoes are cultivated in regions with narrow crop rotations. *S. endobioticum* is often found in such areas, and therefore, cultivation of susceptible varieties is prohibited to contain the spreading of the disease (Baayen et al. 2006). In the ‘Agria’ structure group, 75% of the varieties have *Sen1*: Agria is donor of *Sen1*, and breeders selected P1-resistant progeny. Only ten Agria descendants did not hold the minor allele of PotVar0067008, four of which were resistant (Dione, Excellent, Lady Blanca and Vr-93-146), so we can hypothesise recombination between PotVar0067008 and *Sen1*. Although we showed that PotVar0067008 explained around 69% of the P1 resistance in the GWAS panel, its false-negative rate indicates that there are likely other P1 resistance sources already present in the gene pool that remain to be discovered.

### No other P1 resistance loci identified with the GWAS

To identify other genes involved in P1 resistance, we did GWAS with five *Sen1*-associated markers as cofactor. This new GWAS model did not identify new markers outside the *Sen1* region, suggesting that no other locus is present in the potato genome. Neither could we rediscover the *Sen1*-*4* locus on chromosome *4* (Brugmans et al. 2006). One new chromosome *11* marker PotVar0067876 (at coordinate 3,268,823) emerged as significantly associated with wart resistance. The joint analysis of false positives and false negatives showed nevertheless that PotVar0067876 could not be used to recover false positives or negative predictions from our best marker PotVar0067008. The minor allele of PotVar0067876 was present in simplex in Desiree and Aventra and in duplex in Kuras. The expected segregation ratio would be 3:1 and 11:1 in the A × D and K × A populations, respectively. Therefore, the size of our full-sib populations was too small to (in)validate the significance of the effect of PotVar0067876 on P1 resistance. Another strategy to identify new resistances in the GWAS panel could have been to perform a GWAS in each of the four structure groups. However, the size of these groups did not allow us to follow such a strategy.

### Other P1 resistance loci remain to be identified in the gene pool

The large proportion of false negatives was explored by visual interpretation of a PCoA of varieties without the minor allele of PotVar0067008 (Supplementary Fig. 11). These groups share a specific breeding history along with being frequently resistant to P1. One easy conclusion is to assume that within each group, and another recombined *Sen1* haplotype or source (founder) of resistance was used, having an insufficient allele frequency to pass the MAF threshold or to achieve the statistical power for discovery by GWAS. Specifically, the group of starch varieties was distinguished from the sub-panel. This group contained only Aventra, Festien and Karakter as P1-susceptible varieties. Some of the varieties from this group are known to contain also resistance to other wart pathotypes. This is the case of Allure, Astarte, Avaya, Aviala and Merano (Baayen et al. 2005; NVWA 2018). Possibly, these varieties are resistant to P1 due to other sources than *Sen1.* For example, Merano has the *Sen3* gene which gives resistance to a broader range of pathotypes (Bartkiewicz et al. 2018).

Another interesting observation was that among the 77 varieties from the ‘GB origin’ structure group, only 27 held the minor allele of PotVar0067008. In the remaining 50 varieties, 27 were resistant to P1. It is very likely that (an)other source(s) of P1 resistance, not yet described, are present in the ‘GB’ structure group.

For the structure group of varieties coming from diverse origins (rest), it is difficult to make assumptions about other resistance sources to P1. For Escort, Panda, Royal and Ulme, which are known to be resistant to other pathotypes (NVWA 2018), we can again propose that resistance to P1 is due to another gene conferring resistance to a broader range of pathotypes (such as *Sen3* in Ulme, Prodhomme et al. 2019; Bartkiewicz et al. 2018). For other varieties, which are only resistant to P1, the resistance could still come from *Sen1* if they recombined between PotVar0067008 and *Sen1* or from another locus not yet identified.

### Limitations to the identification of markers with complete diagnostic value

PotVar0067008 is the most closely linked marker to *Sen1* that we identified with the GWAS. However, its diagnostic value was not optimal as it showed a false-positive rate of 4% and a false-negative rate of 24%. An explanation for false positives can be recombinations which occurred in some varieties between the marker and *Sen1* or background dependency of the *Sen1* gene. The explanation for false negatives can be recombinations between the marker and *Sen1* as well, but also the presence of other alleles or other loci bringing resistance to P1 in the panel. With the genotypic data we used in the GWAS, we could not identify markers with a better diagnostic value than PotVar0067008 or PotVar0066530 for which recombinations with *Sen1* have been observed in the biparental populations.

When comparing the physical coordinates of the most significant markers from our GWAS study with genetic positions of *Sen 1* from the literature, and from our validation population, we noticed that we did not achieve to identify strongly associated SNPs north of PotVar0066530 and *Sen1*. Was our GWAS analysis hampered by ascertainment bias or by insufficient numbers of markers, or a strong decay of LD? Ascertainment bias is excluded because the SNP discovery panel includes 83 varieties of which many have *Sen1*. When evaluating marker sufficiency and their distribution, it appeared that 3104 SNP markers have been described in the first 4 Mb of chromosome *11* (Uitdewilligen et al. 2013) and that a subset of 288 SNPs were placed on the SolSTW array (Vos et al. 2015). Their distribution depends on the baits designed for targeted resequencing and is shown in Supplementary Fig. 12. It appears that no markers were designed between 814,888 bp and 1,416,133 bp meaning that we could not identify markers flanking north of the TNL cluster C76 (located between 1,333,729 bp and 1,589,104 bp, Jupe et al. 2013). No markers were designed either between 1,417,043 bp and 1,952,061 bp hampering the design of diagnostic markers closer to *Sen1* (Supplementary Fig. 12). Moreover, from a region at 1.42 Mb, comprising 146 SNPs, only four SNPs were selected for the SNP array. This uneven coverage of designed SNP markers in the *Sen1* region in the Uitdewilligen data set could be the primary reason that we did not identify SNP markers with better association than PotVar0067008. By graphical genotyping, we also observed that no marker specific to the *Sen1* haplotype could be identified north to 2,7 Mb. The lack of haplotype specificity of the markers north to PotVar0066530 hampered the identification of markers with complete diagnostic value.

## Conclusion

This study illustrates that pre-existing genotypic data and a collection of historical phenotypic data allowed to screen potato germplasm for genes involved in P1 resistance and to identify the haplotype-specific marker PotVar0067008 linked to *Sen1*, with a false-positive rate of only 4%. Because this SNP was identified using a GWAS approach, we know that the utility of this KASP marker can be extrapolated to a wide genetic background. We conclude that the *Sen1* locus, first published by Hehl et al. (1999), is the most frequent source of P1 resistance, but other less frequent alleles or loci remain to be identified. In general, marker-assisted breeding demands for SNPs without false positives and false negatives. This paper shows reasons why this is not always feasible. First, we could not trace founders of *Sen1* and suspect a lack of identity by descent of *Sen1* bearing haplotypes. Second, the raw marker data describe more than a century of potato breeding and clones with recombinant haplotypes were identified, which illustrates the process of decay of LD between flanking markers and *Sen1*.

## Electronic supplementary material

Below is the link to the electronic supplementary material.

Supplementary material 1 (DOCX 38 kb)

Supplementary material 2 (PPTX 40168 kb)

Supplementary material 3 (XLSX 12212 kb)

Supplementary material 4 (XLSX 27 kb)

Supplementary material 5 (XLSX 53 kb)

Supplementary material 6 (XLSX 16 kb)
