# Supplementary material for: Distribution of P1(D1) wart disease resistance in potato germplasm and GWAS identification of haplotype-specific SNP markers
Source: Theor Appl Genet. 2020 Feb 11;133(6):1859–71. doi: 10.1007/s00122-020-03559-3 (PMC7237394; doi:10.1007/s00122-020-03559-3)
Supplement: Supplementary file 1 — Supplementary material 1 (DOCX 38 kb) [file 122_2020_3559_MOESM1_ESM.docx]

# Supplementary figures

**Supplementary figure 1**

Distribution of the pathotype 1 resistance scores in the GWAS panel (right stacked histogram). Colours are given in function of the year of release of the variety. The scores ranged from 0 to 11 as we calculated for each variety a mean score corrected for the source effect. For each resistance category( [0-2], ]2-4], ]4-6], ]6-8], ]8-11]), the varieties origin is given (left table). AUT = Austria, BRD = Bundesrepublik Deutschland (West Germany), CAN = Canada, DDR = Deutsche Demokratische Republik (East Germany), DEN = Denmark, ESP = Spain, FRA = France, GB = Great Britain, GER = Germany, HOL = Netherlands, IRL = Ireland, POL = Poland, SWE = Sweden.

**Supplementary figure 2**

Boxplots of the distribution of the pathotype 1 resistance scores in function of the four structure groups identified with the PCoA and delimited with the Structure analysis.

**Supplementary figure 3**

Distribution of the residues of the GWAS models. Three GWAS models have been tested: a naive model with no correction (1), a corrected model with 30 PCOs included as a fixed effect (2) and a corrected model with 30 PCOs and 5 co-factors (PotVar0067008, PotVar0067303, PotVar0066243, solcap_snp_c2_13431, PotVar0066434) as fixed effects (3). In (a) are shown the histograms of the residues distribution and in (b) the qq-plots. When including the chromosome 11 uncorrelated most significant markers, the distribution of the residues is very close to normality.

**Supplementary figure 4**

Quantile-quantile plot (qq-plot) of the GWAS *p*-values (-log_10_(*p*)) with (green) or without (blue) PCoA correction. The qq-plot shows the observed distribution of association test statistics compared to the expected distribution of associations among the 10,968 SNPs used in the analysis. Including a PCoA correction as a fixed effect in the GWAS model decreases the deviation from the X = Y line (red) for the lower part of the curve, decreasing the chance to find false associations due to the structure confounding factor.

**Supplementary figure 5**

Distribution of the resistance means (2016 + 2017) scored with the Glynne-Lemmerzahl method (a and b) and with the Spieckermann method (c and d) in the AxD (a and c) and the KxA (b and d) populations. Black bars represent the number of individuals holding the minor allele of PotVar0066530. The threshold between R and S descendants is represented with a dashed line.

**Supplementary figure 6**

Graphical genotyping in the 135K dataset developed by Uitdewilligen *et al*., (2013), with the following operators: allele frequency between 12 and 24%, present in Arran Pilot, absent in Black 1256, Ackersegen, Y 66-13-636 and Yam, an haploblock of 54 SNPs was identified including Potvar0067008.

**Supplementary figure 7**

Graphical genotyping in the 135K dataset developed by Uitdewilligen *et al*. (2013), with the following operators: MAF > 30%, absent from the susceptible varieties Bintje, Industrie, and Arran Chief, present in the resistant variety Arran Pilot, 31 SNPs in high LD with PotVar0066243 were identified.

**Supplementary figure 8**

Effect of the *Sen1* markers on pathotype 1 resistance. (a) Effect of PotVar0067008 on P1 resistance in the GWAS panel. (b) Effect of PotVar0066530 in the validation populations on P1 resistance assessed with the Spieckermann method (average 2016 +2017) and (c) with the Glynne-Lemmerzahl method (average 2016 +2017). 0, 1, 2 and 3 represent the dosage of the marker (absent, simplex, duplex and triplex respectively).

**Supplementary figure 9**

(a) Dosage of PotVar0067008 minor allele in function of the structure groups and (b) proportion of each structure group with the minor allele of PotVar0067008 in nulliplex, simplex, duplex or triplex.

**Supplementary figure 10**

Miami plot of the GWAS of pathotype 1 resistance using 330 potato varieties. The PCoA correction and five chromosome 11 markers as cofactors is shown in the upper part of the plot (in orange). The PCoA correction alone is shown in the lower part of the plot (in green). The association (-/+log_10_(*p*)) of each SNP with pathotype 1 resistance is represented. The x axis represents the physical position of each SNP on the 12 potato chromosomes (u corresponds to unanchored and chloroplastic markers). The red horizontal lines correspond to the significance threshold calculated with the Li and Ji method (-log_10_(*p*) = 4.7).

**Supplementary figure 11**

The structure of the sub-panel of 144 varieties, which do not hold the minor allele of PotVar0067008. Still a large proportion of the varieties are resistant to pathotype 1. The PCoA was performed with a random subset of 1,000 SNPs. Each variety is represented by a pseudo-colour that scales with the level of resistance to pathotype 1 (dark colour (P1 predicted mean < 6) indicates highly susceptible varieties). The shapes of the dots indicate the structure group affiliation of each variety.

**Supplementary figure 12**

Physical distribution of PotVar SNPs in the first 4 Mb of chromosome *11*. The volume of 3,104 SNPs identified by targeted resequencing is shown in blue (Uitdewilligen *et al*. 2013). Only 288 SNPs (shown in red) were deployed on the SolSTW SNP array (Vos *et al*. 2015). The position of candidate *R* gene clusters are indicated with green boxes (Jupe *et al*. 2013).

# Supplementary tables

**Supplementary table 1**

Re-phenotyping results of the 12 varieties for which preliminary analysis showed inconsistencies between the markers prediction and the phenotype. The varieties were re-assessed in spring 2016 with the Spieckermann (9 eye-pieces) and the Glynne-Lemmerzahl (5 eye-pieces) methods. Mean scores are given in the table. Deodara was used as a susceptible control. The phenotypic dataset was corrected according to these results and historical data records were discarded.

| **Variety** | **Spieckermann** | **Glynne-Lemmerzahl** |
| --- | --- | --- |
| **Agria** | 10 | 9.2 |
| **Belana** | 10 | 8.8 |
| **Bellanova** | 10 | 8.8 |
| **Berber** | 10 | 8.2 |
| **Caruso** | 10 | 9.2 |
| **Cherie** | 10 | 9.8 |
| **Elisabeth** | 10 | 9.6 |
| **Forza** | 9.8 | 9.8 |
| **Kastelli** | 10 | 9.7 |
| **Lady Jo** | 10 | 9 |
| **Laura** | 10 | 9.2 |
| **Starga** | 10 | 8.6 |
| **Deodara** | 6.9 | 2.2 |

**Supplementary table 2**

Primers of the KASP markers used to validate the GWAS results in the two independent bi-parental populations.

| Marker | Forward primer (R allele) (5'- 3') | Forward primer (S allele) (5'- 3') | Reverse primer (5'- 3') |
| --- | --- | --- | --- |
| PotVar0066530 | ATATTTTAGTGTGTTTCTCTGTGCTGC | ATATTTTAGTGTGTTTCTCTGTGCTGG | GACAAAGAAAGCAGTAGCACTTTCTCATT |
| PotVar0067008 | GGCATTAAATTCCTTAAAACTCTCAGCG | GCATTAAATTCCTTAAAACTCTCAGCA | GGAAAGCTTTTTTGTTACAAGGTGGAGAT |
| solcap_snp_c1_2314 | CTTCATCTCTTTCTTTCATAACTCTAGC | TCATCTCTTTCTTTCATAACTCTAGT | GATGAGGAGATTTTGCAACTTAAGGATCTA |
| PotVar0106272 | CCAATTCACCAGGAAGAACCAATCT | CAATTCACCAGGAAGAACCAATCG | ACTTCACGGGAAATTCAAACTGCTGTT |
| PotVar0105904 | GAGCAAGTAAGCCAATGCCAATTCAT | AGCAAGTAAGCCAATGCCAATTCAG | GCCTAGTACTTCTCGTGTTGTTTTCAAAA |

**Supplementary table 3**

Significantly associated marker in the GWAS structure and cofactors corrected model results. The chromosome, the physical coordinate at PGSC v4.03 (PGSC, 2011), the -log_10_(*p*) value, the estimation of the minor allele effect in the GWAS panel and its allele frequency (MAF) are given. The false positives rate (FPR), which is the proportion of individuals being susceptible whereas they have the marker (varieties having a resistance score < 8 are considered as being susceptible), and the false negative rate (FNR), which is the proportion of individuals being resistant whereas they lack the marker, are also given.

| SNP | Chromo-some | Position (bp) | *p*-value | Effect | MAF | FPR | FNR |
| --- | --- | --- | --- | --- | --- | --- | --- |
| PotVar0067876 | 11 | 3,268,823 | 9.14E-06 | 1.08 | 0.23 | 0.16 | 0.20 |

# Supplementary files

**Supplementary file 1**

First sheet: Overview of the 330 potato varieties of our GWAS panel with their year of market release, their country of origin, the structure group to which the variety was assigned, the phenotypic score obtained from the data in Additional file 3. This score was used to classify varieties into resistant or susceptible. The significantly associated markers are shown with their coordinates (PGSC v4.03 (PGSC, 2011)) and their allele dosage. Our interpretation on concordance between marker PotVar0067008 and resistance is shown in the last columns where false positives are susceptible varieties which hold the minor allele of PotVar0067008 and false negatives are resistant varieties which do not hold the minor allele of PotVar0067008.

Second sheet: Genotypic data input used in the GWAS. Markers randomly selected with replacement for the vanRaden kinship calculation are indicated in column B.

**Supplementary file 2**

Structure group membership of each variety according to the STRUCTURE results for K = 4. These results were used to describe the distribution of P1 resistance and the distribution of *Sen1* in the different groups.

**Supplementary file 3**

Overview of the historical sources that provided data on wart disease resistance in the 330 potato varieties used in our GWAS panel. These historical sources include documents for National listing where VCU data were recorded, marketing brochures from commercial breeders as well as scientific publications. Many sources use different scales to describe the level of wart disease resistance. Qualitative data R and S were converted into 10 and 1 respectively. This allowed compilation of the next tab sheet showing all records used to calculate a single phenotypic score using REML.

**Supplementary file 4**

Phenotypic and genotypic observations collected across years and assays from the offspring of the AxD and KxA validation populations, along with the Mendelian classification into R and S for linkage analysis and map construction.
