# Supplementary material for: Distribution of P1(D1) wart disease resistance in potato germplasm and GWAS identification of haplotype-specific SNP markers
Source: Theor Appl Genet. 2020 Feb 11;133(6):1859–71. doi: 10.1007/s00122-020-03559-3 (PMC7237394; doi:10.1007/s00122-020-03559-3)
Supplement: Supplementary file 2 — Supplementary material 2 (PPTX 40168 kb) [file 122_2020_3559_MOESM2_ESM.pptx]

## Slide 1
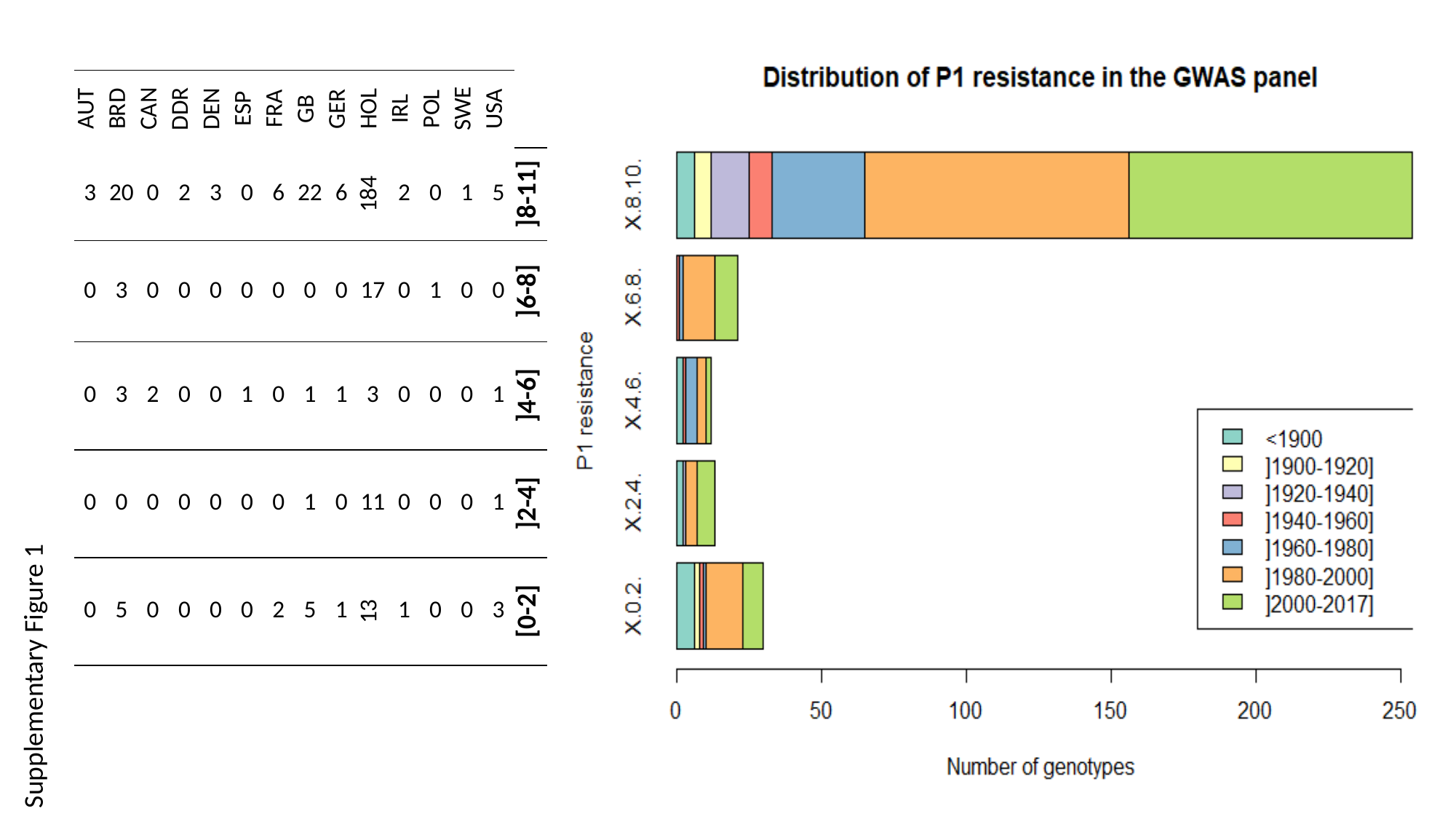

| AUT | BRD | CAN | DDR | DEN | ESP | FRA | GB | GER | HOL | IRL | POL | SWE | USA | |
| --- | --- | --- | --- | --- | --- | --- | --- | --- | --- | --- | --- | --- | --- | --- |
| 3 | 20 | 0 | 2 | 3 | 0 | 6 | 22 | 6 | 184 | 2 | 0 | 1 | 5 | ]8-11] |
| 0 | 3 | 0 | 0 | 0 | 0 | 0 | 0 | 0 | 17 | 0 | 1 | 0 | 0 | ]6-8] |
| 0 | 3 | 2 | 0 | 0 | 1 | 0 | 1 | 1 | 3 | 0 | 0 | 0 | 1 | ]4-6] |
| 0 | 0 | 0 | 0 | 0 | 0 | 0 | 1 | 0 | 11 | 0 | 0 | 0 | 1 | ]2-4] |
| 0 | 5 | 0 | 0 | 0 | 0 | 2 | 5 | 1 | 13 | 1 | 0 | 0 | 3 | [0-2] |
Supplementary Figure 1

## Slide 2
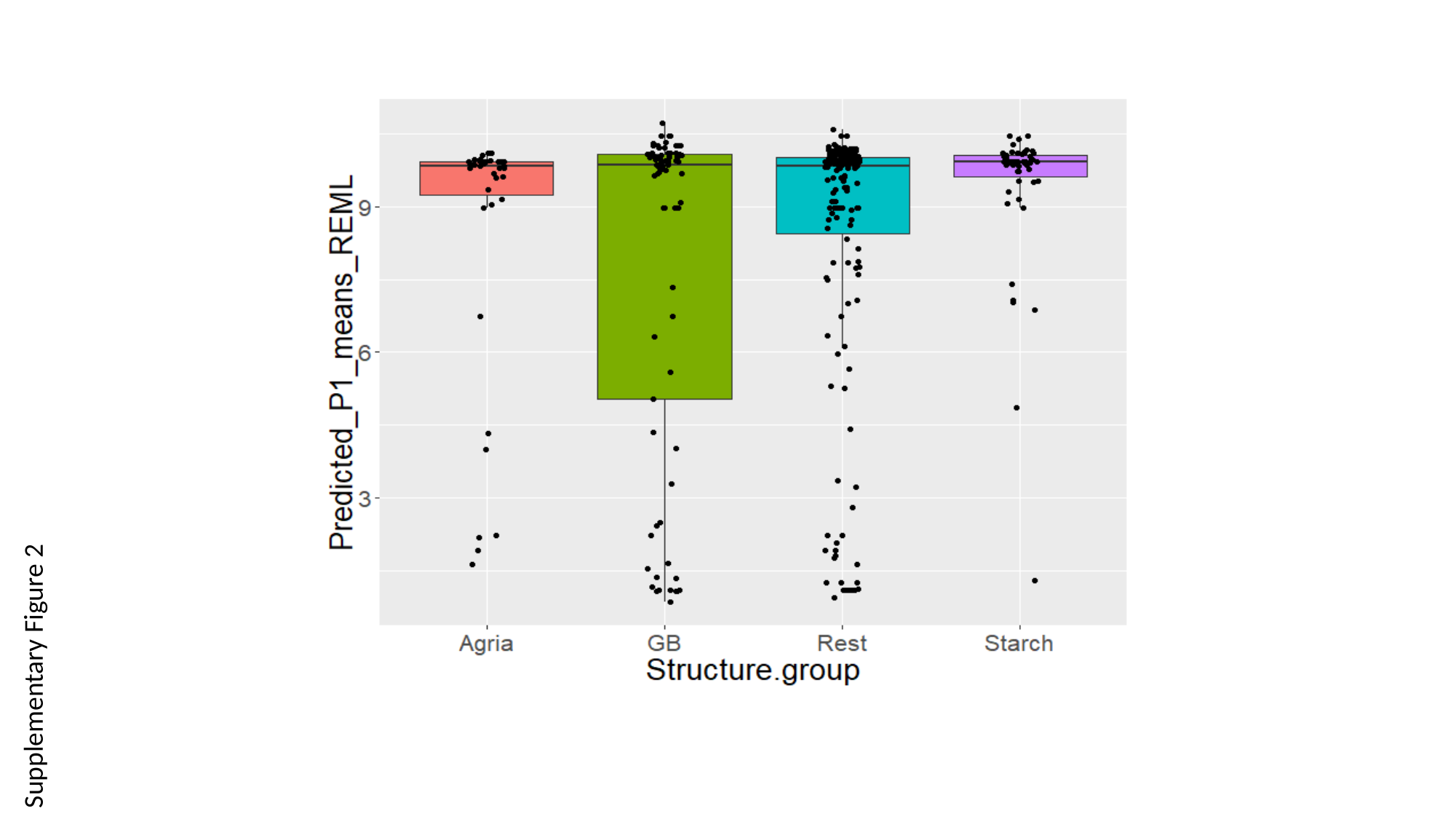

Supplementary Figure 2

## Slide 3
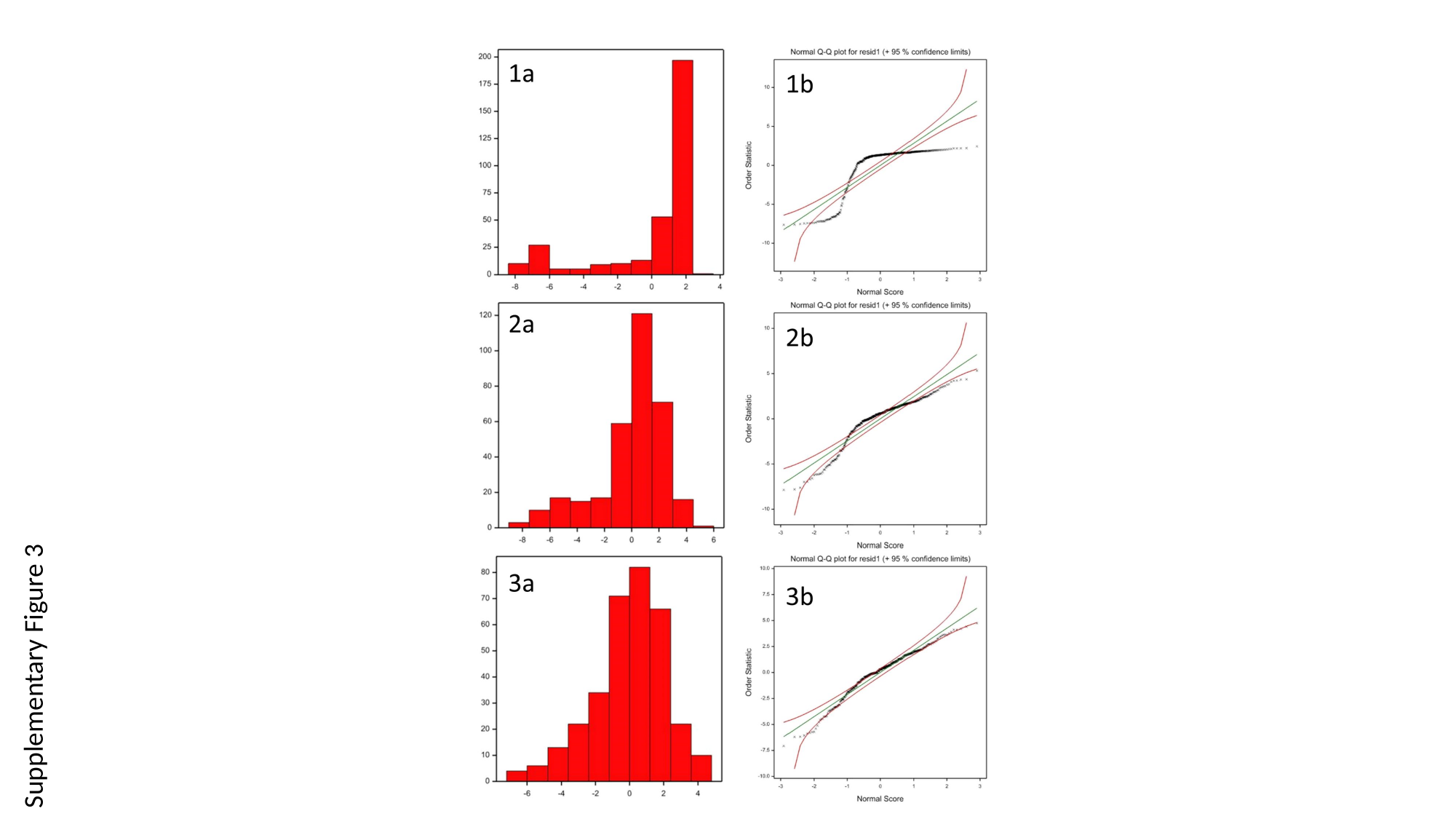

Supplementary Figure 3

## Slide 4
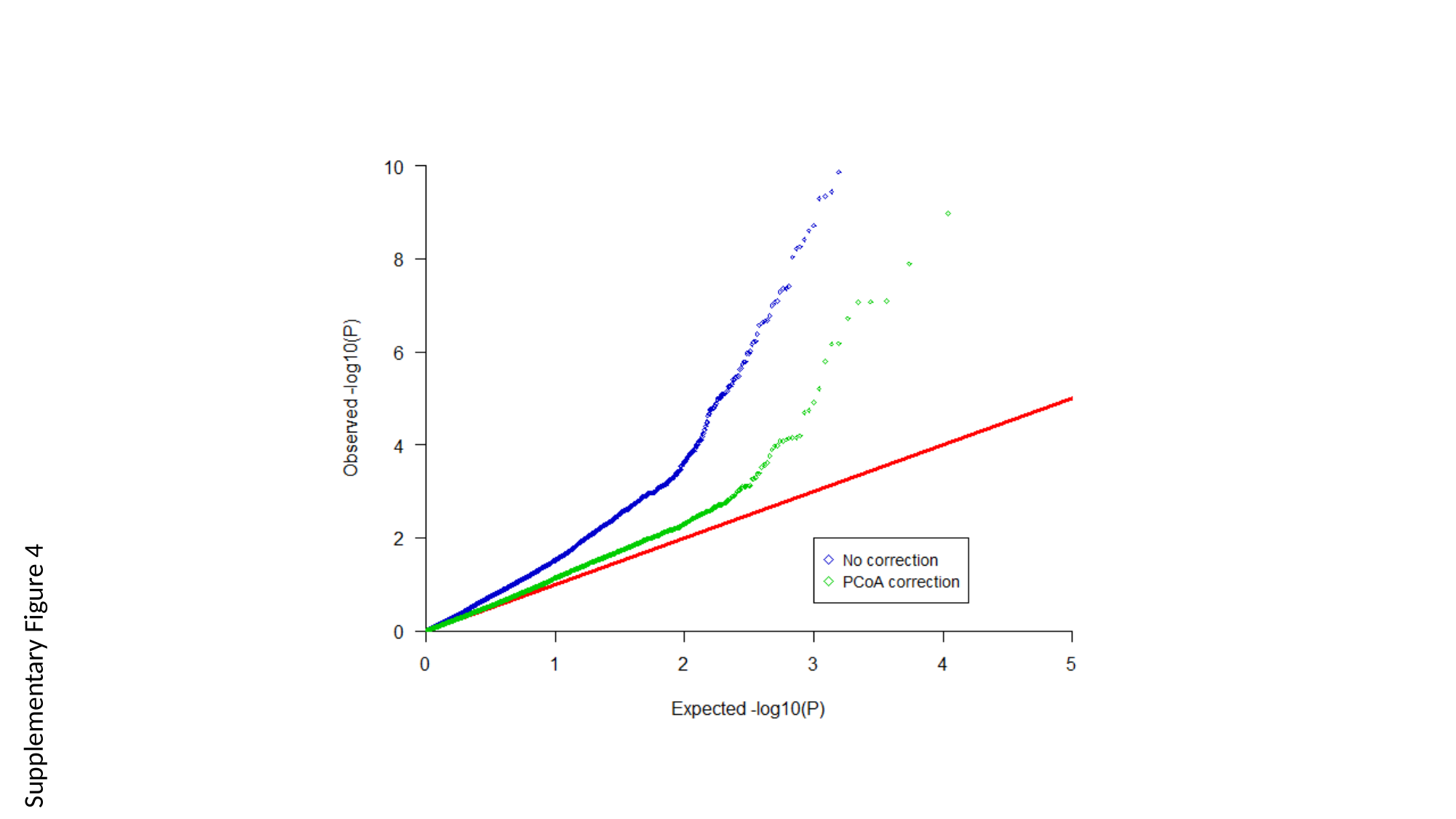

Supplementary Figure 4

## Slide 5
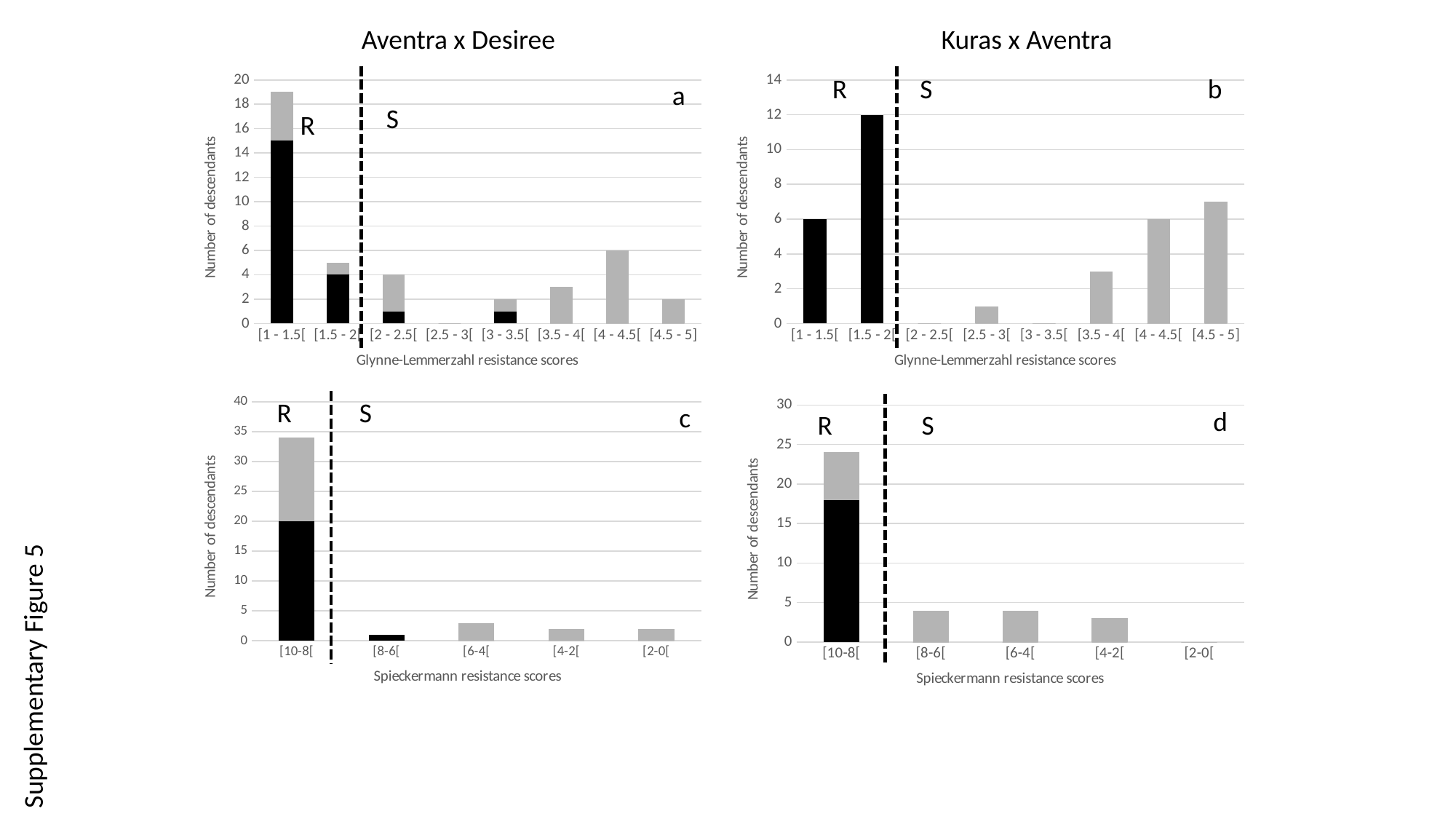

Aventra x Desiree
Kuras x Aventra
### Chart
| Category | R allele | S allele |
|---|---|---|
| [1 - 1.5[ | 15.0 | 4.0 |
| [1.5 - 2[ | 4.0 | 1.0 |
| [2 - 2.5[ | 1.0 | 3.0 |
| [2.5 - 3[ | 0.0 | 0.0 |
| [3 - 3.5[ | 1.0 | 1.0 |
| [3.5 - 4[ | 0.0 | 3.0 |
| [4 - 4.5[ | 0.0 | 6.0 |
| [4.5 - 5] | 0.0 | 2.0 |
### Chart
| Category | R allele | S allele |
|---|---|---|
| [1 - 1.5[ | 6.0 | 0.0 |
| [1.5 - 2[ | 12.0 | 0.0 |
| [2 - 2.5[ | 0.0 | 0.0 |
| [2.5 - 3[ | 0.0 | 1.0 |
| [3 - 3.5[ | 0.0 | 0.0 |
| [3.5 - 4[ | 0.0 | 3.0 |
| [4 - 4.5[ | 0.0 | 6.0 |
| [4.5 - 5] | 0.0 | 7.0 |a
R
### Chart
| Category | R allele | S allele |
|---|---|---|
| [10-8[ | 20.0 | 14.0 |
| [8-6[ | 1.0 | 0.0 |
| [6-4[ | 0.0 | 3.0 |
| [4-2[ | 0.0 | 2.0 |
| [2-0[ | 0.0 | 2.0 |
### Chart
| Category | R allele | S allele |
|---|---|---|
| [10-8[ | 18.0 | 6.0 |
| [8-6[ | 0.0 | 4.0 |
| [6-4[ | 0.0 | 4.0 |
| [4-2[ | 0.0 | 3.0 |
| [2-0[ | 0.0 | 0.0 |Supplementary Figure 5

## Slide 6
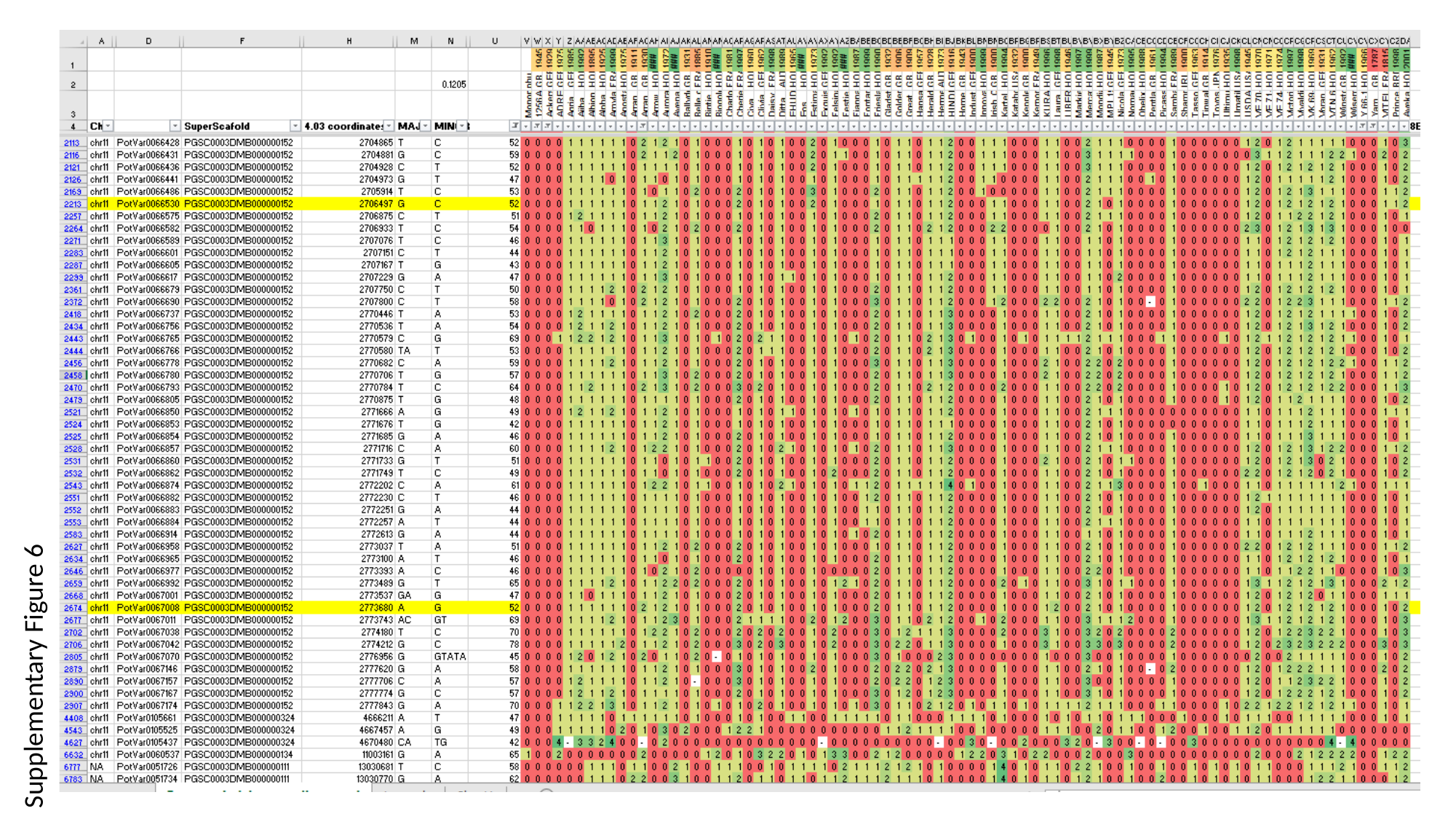

Supplementary Figure 6

## Slide 7
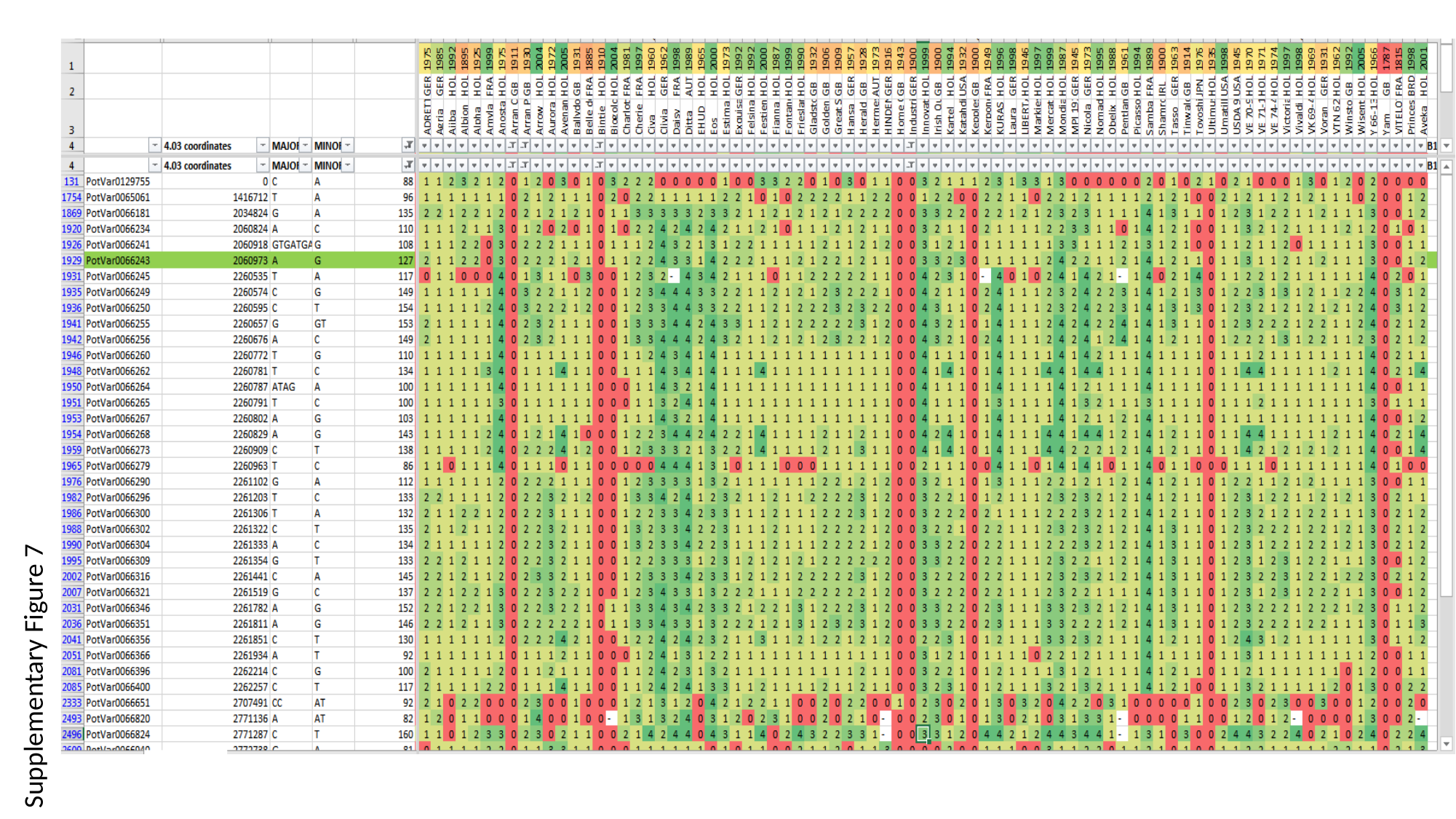

Supplementary Figure 7

## Slide 8
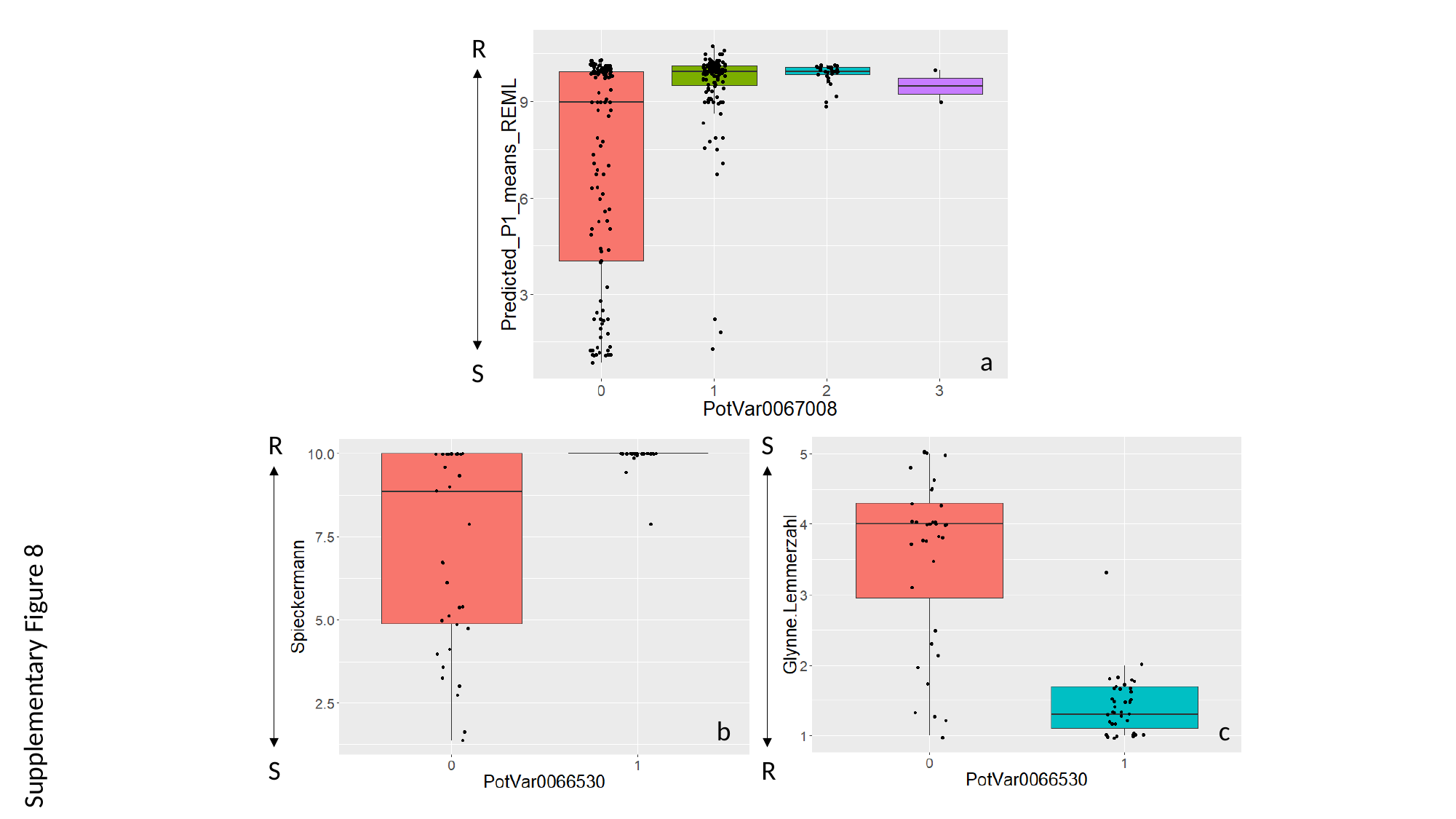

R
a
S
R
S
Supplementary Figure 8
b
c
S
R

## Slide 9
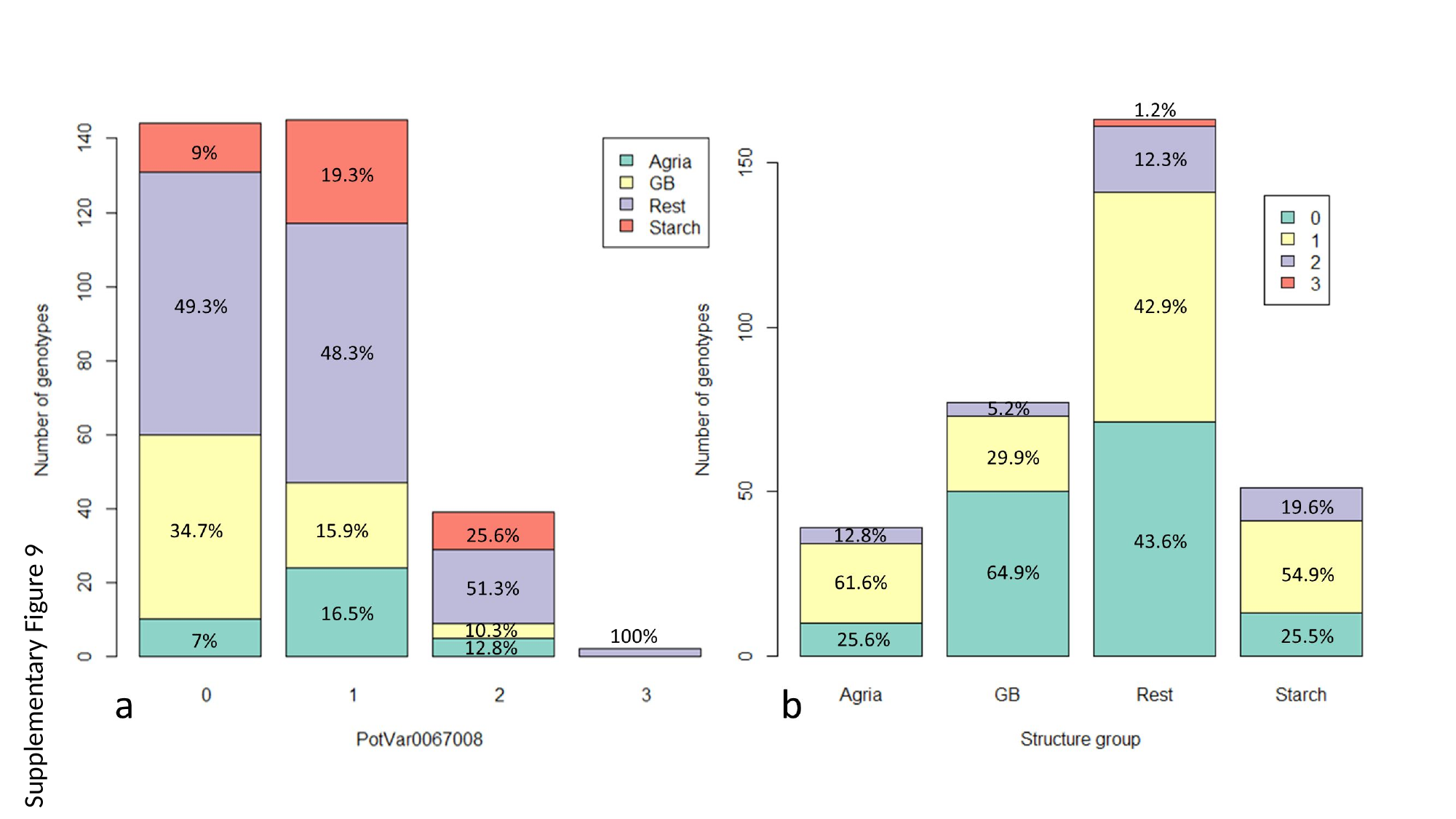

Supplementary Figure 9

## Slide 10
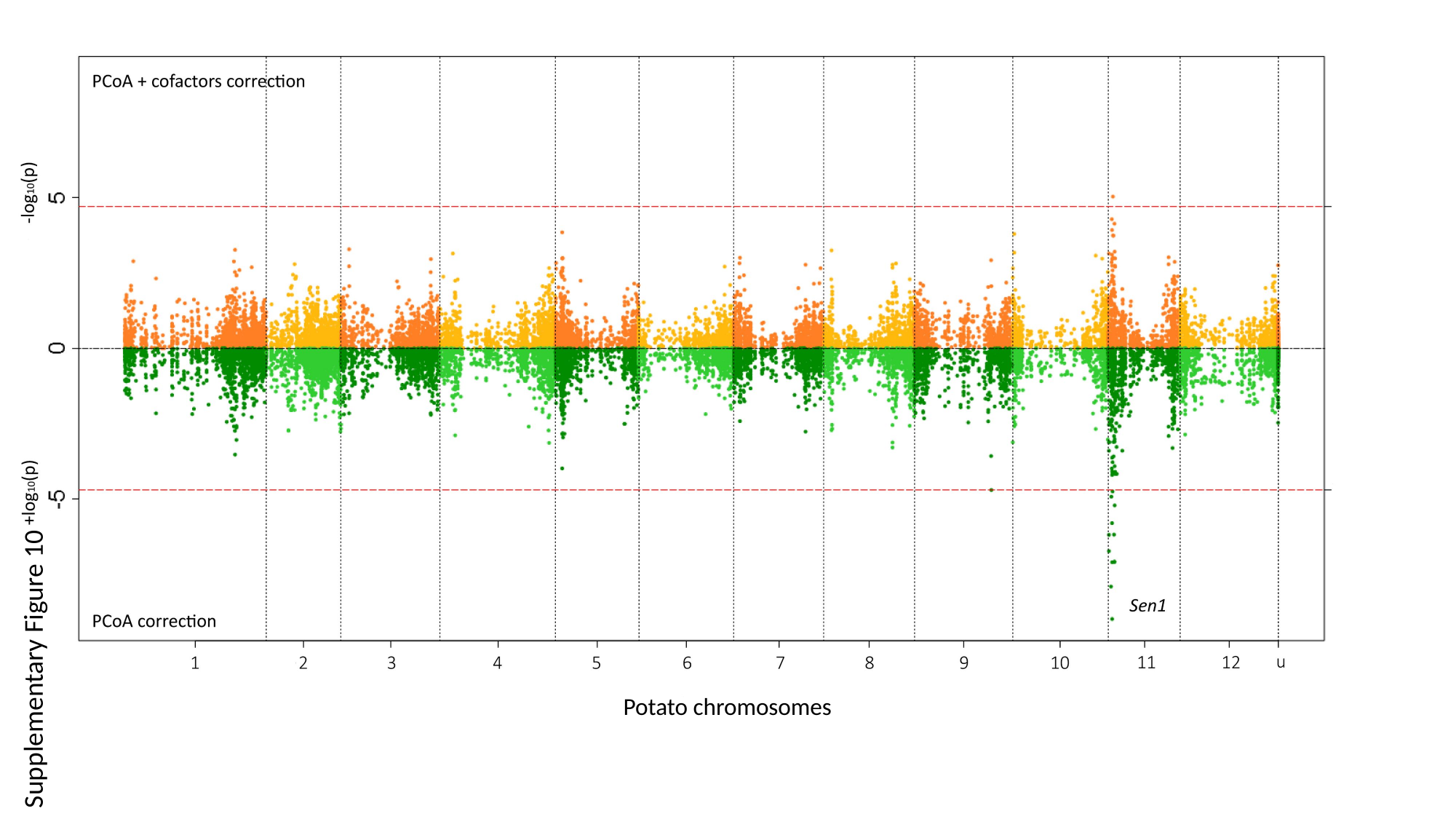

Supplementary Figure 10
Potato chromosomes

## Slide 11
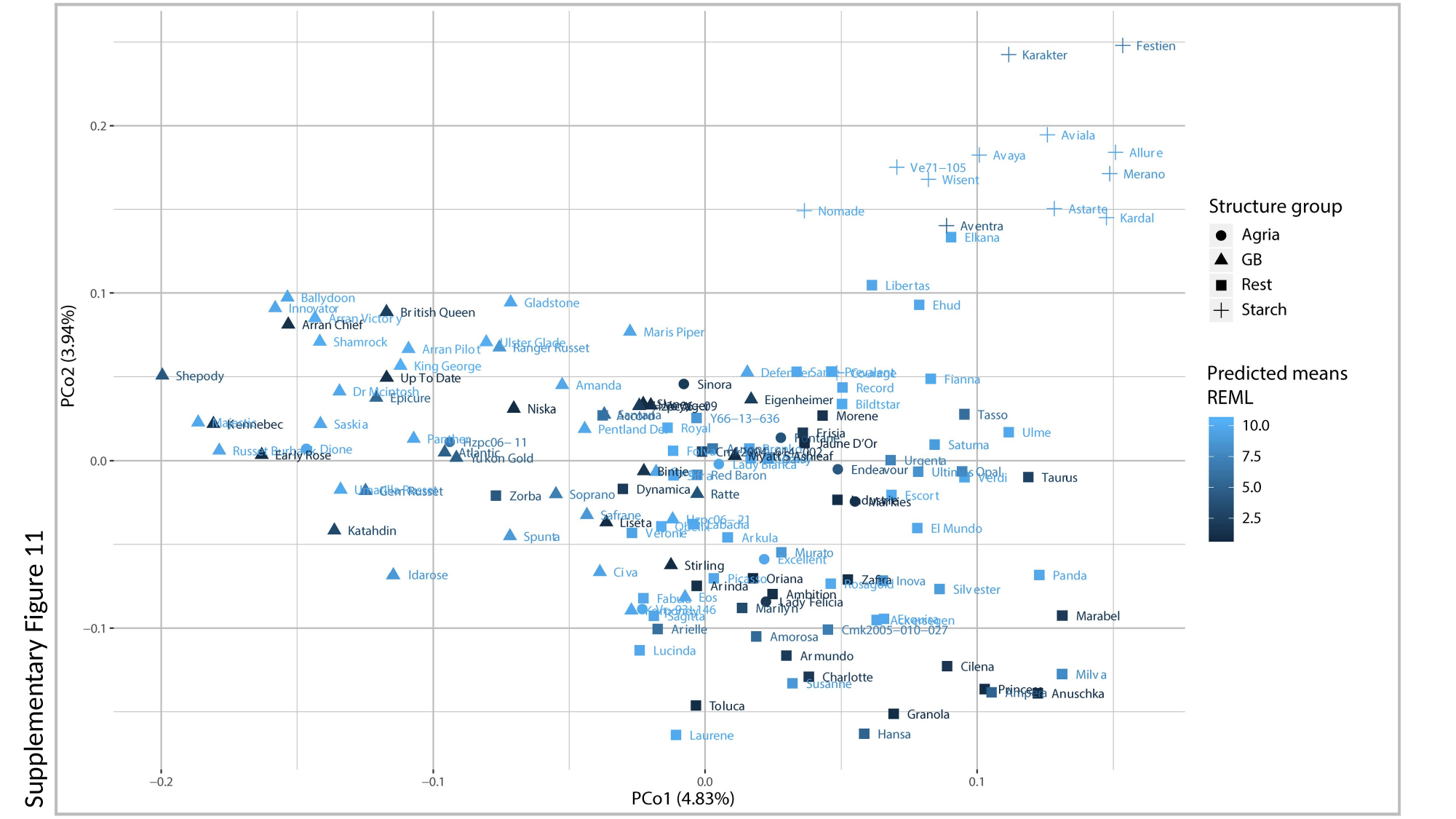

Supplementary Figure 11

## Slide 12
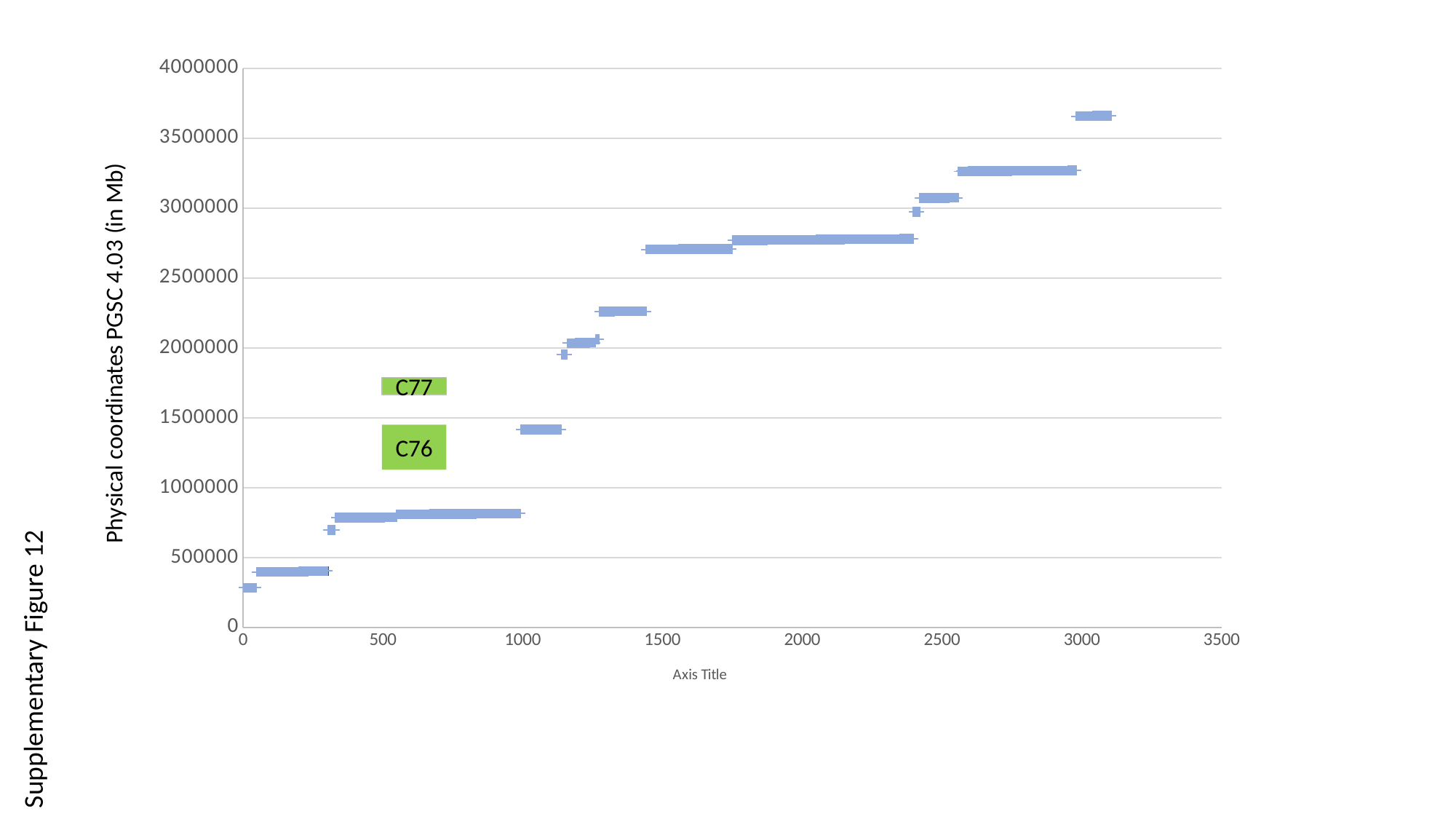

### Chart
| Category | | |
|---|---|---|C77
C76
Supplementary Figure 12
